# Fine Tuning Muscarinic Acetylcholine Receptor Signaling Through Allostery and Bias

**DOI:** 10.3389/fphar.2020.606656

**Published:** 2021-01-29

**Authors:** Emma T. van der Westhuizen, K. H. Christopher Choy, Celine Valant, Simon McKenzie-Nickson, Sophie J. Bradley, Andrew B. Tobin, Patrick M. Sexton, Arthur Christopoulos

**Affiliations:** ^1^ Drug Discovery Biology, Monash Institute for Pharmaceutical Research, Monash University, Parkville, VIC, Australia; ^2^ Centre for Translational Pharmacology, Institute of Molecular Cell and Systems Biology, University of Glasgow, Glasgow, United Kingdom

**Keywords:** muscarinic receptor, allosteric modulation, Alzheimer’s disease, schizophrenia, biased agonism, biased allostery

## Abstract

The M_1_ and M_4_ muscarinic acetylcholine receptors (mAChRs) are highly pursued drug targets for neurological diseases, in particular for Alzheimer’s disease and schizophrenia. Due to high sequence homology, selective targeting of any of the M_1_-M_5_ mAChRs through the endogenous ligand binding site has been notoriously difficult to achieve. With the discovery of highly subtype selective mAChR positive allosteric modulators in the new millennium, selectivity through targeting an allosteric binding site has opened new avenues for drug discovery programs. However, some hurdles remain to be overcome for these promising new drug candidates to progress into the clinic. One challenge is the potential for on-target side effects, such as for the M_1_ mAChR where over-activation of the receptor by orthosteric or allosteric ligands can be detrimental. Therefore, in addition to receptor subtype selectivity, a drug candidate may need to exhibit a biased signaling profile to avoid such on-target adverse effects. Indeed, recent studies in mice suggest that allosteric modulators for the M_1_ mAChR that bias signaling toward specific pathways may be therapeutically important. This review brings together details on the signaling pathways activated by the M_1_ and M_4_ mAChRs, evidence of biased agonism at these receptors, and highlights pathways that may be important for developing new subtype selective allosteric ligands to achieve therapeutic benefit.

## Introduction

### The Central Cholinergic System

**FIGURE 1:**
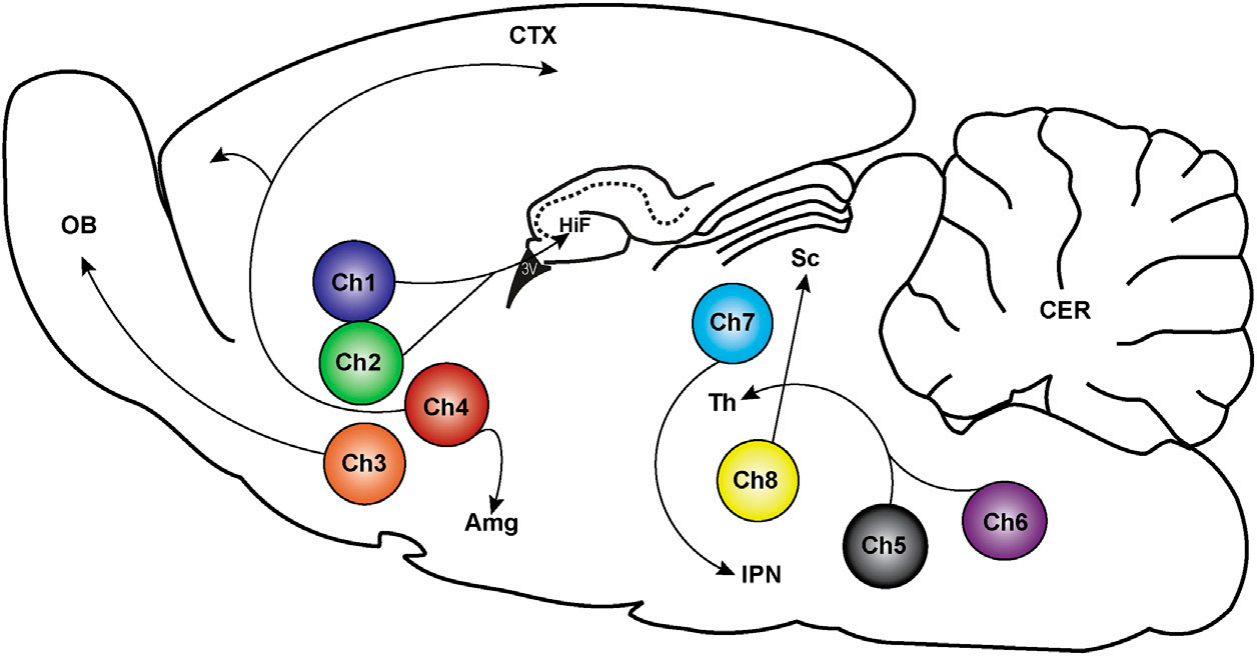
Location of cholinergic neurons and their projections in the rodent brain. Cholinergic neurons exist in eight distinct clusters labeled Ch1-Ch8. Ch1 and Ch2 are located in the medial septum and the diagonal band of Broca and send projections to the hippocampus (HiF), medial cortex, and thalamic nuclei (Th). The Ch3 cluster is in the diagonal band of Broca with projections to the olfactory bulb (OB). Ch4 neurons are in the nucleus basalis magnocellularis contains, which project to the cortex (CTX) and the amygdala (Amg). Ch5 and Ch6 are in the pedunculopontine tegmental nucleus and laterodorsal tegmental nucleus, respectively, which project to the thalamus (Th), pontine reticular formation, ventral midbrain, ventral tegmental area and to the substantia nigra. Ch7 neurons are in the habenula with projections to the interpeduncular nucleus (IPN). Ch8 are in the parabigeminal nucleus that project to the superior colliculus (Sc) (Thiele 2013; Li et al., 2018).

Acetylcholine (ACh) is a neurotransmitter that plays a vital role in central nervous system (CNS) function. ACh is synthesized from the nutrient, choline, by the enzyme, cholineacetyltransferease and subsequently stored in intracellular vesicles in cholinergic projection neurons and cholinergic interneurons (Amenta and Tayebati, 2008). The cell bodies of cholinergic neurons reside in eight distinct clusters, which are named Ch1-Ch8. These clusters send projections to innervate distinct regions of the brain, as depicted in Figure 1 (Thiele 2013; Allaway and Machold, 2017; Li et al., 2018). Besides the main clusters of cholinergic projection neurons, cholinergic signaling also occurs in other local networks of neurons. One such cluster is located within the basal ganglia (striatum, caudate-putamen and globus pallidus), which is without external cholinergic inputs. Another cluster is found in the cortex and contains many cholinergic interneurons (Lanciego et al., 2012; Li et al., 2018). The central cholinergic signaling system contributes to many critical brain functions, including arousal, attention, learning and memory, sensory perception, motor function, sleep, nociception, motivation, reward, mood, psychosis and neuroplasticity (Thiele 2013; Hangya et al., 2015). At the cellular level, ACh regulates neuronal functions such as cell excitability and firing, neurotransmitter release and synaptic plasticity through its actions at pre- and post-synaptic acetylcholine receptors (Picciotto et al., 2012; Thiele 2013).

### Receptors Responding to Acetylcholine

ACh signaling is triggered through activation of two major classes of cell-surface receptor proteins, the nicotinic ACh receptors (nAChRs; ligand-gated ion channels) and the muscarinic AChRs (mAChRs; G protein-coupled receptors; GPCRs). Both the nAChRs and the mAChRs are expressed in the CNS and in the periphery (Gotti et al., 2009; Chatzidaki and Millar, 2015) and participate in neuronal signal transduction events. The nAChRs are located both pre- and post-synaptically on neurons. They are important for fast synaptic transmission, allowing the passage of sodium, potassium and calcium ions (Gotti et al., 2009). Within the mAChR family, there are five subtypes that are aptly named M_1_–M_5_. All five subtypes of mAChRs are expressed in both the CNS and within peripheral tissues.

#### Distribution of mAChR Subtypes in the Brain

Quantitative immunoprecipitation, immunocytochemistry and electron microscopy techniques identified high expression levels of M_1_ mAChR in the striatum, prefrontal cortex, hippocampus and many neocortical regions (Buckley et al., 1988; Levey et al., 1991; Mrzljak et al., 1993; Vilaro et al., 1993; Flynn et al., 1995; Levey, 1996). The M_1_ mAChR is predominantly post-synaptic, playing important roles in the control of glutamatergic neurotransmission (Buckley et al., 1988; Levey et al., 1991; Levey, 1996). The M_2_ and M_4_ mAChRs are co-expressed with the M_1_ mAChR in forebrain regions, albeit at much lower levels (Buckley et al., 1988; Levey et al., 1991; Levey, 1996). The M_4_ mAChR is the most abundant subtype in the striatum, caudate and putamen (Buckley et al., 1988; Levey et al., 1991; Levey, 1996), with both pre- and post-synaptic expression, regulating dopamine release in the striatum (Foster et al., 2016; Moehle et al., 2017; Klawonn et al., 2018; Nair et al., 2019). The M_2_ mAChR is the predominant subtype in the basal forebrain, and is a pre-synaptic autoreceptor that sends signals to stop the release of ACh into synaptic clefts (Levey et al., 1991; Levey, 1996). In contrast, the M_3_ and M_5_ subtypes are expressed at low levels within the brain (Levey et al., 1991; Levey, 1996). Although all of these subtypes play important functional roles in central cholinergic signaling, the focus for this review will be on the M_1_ and M_4_ mAChRs, which have recently emerged as promising drug targets for the treatment of cognitive and behavioral symptoms of neurodegenerative and neurodevelopmental disorders.

## Neurological Disorders Associated With Cholinergic System Dysfunction

Increasing evidence indicates that an imbalance in cholinergic signaling is a major contributor to the prevailing symptoms of many neurological diseases, including Alzheimer's disease, Parkinson's disease, schizophrenia, depression and bipolar disorder; either directly or as a result of modulating other important neurotransmitters (Langmead et al., 2008; Carruthers et al., 2015; van Enkhuizen et al., 2015; Foster et al., 2016; Nair et al., 2019).

### Alzheimer’s Disease

Alzheimer's disease is a neurodegenerative disease that presents as a progressive decline in memory. It is characterised by the accumulation of amyloid-β protein plaques in neurons and hyperphosphorylation of microtubule associated Tau proteins (Kar et al., 2004; Thathiah and De Strooper, 2011; Hartl et al., 2015). Accumulation of protein aggregates in neurons, results in the dysregulation of multiple neurotransmitter systems. The cholinergic system is one of the key transmitter systems that are affected, with degeneration of the cholinergic neurons of the basal forebrain reducing the amount of ACh available for neurotransmission. In post-mortem Alzheimer's disease brain tissue, the expression levels of the M_2_ mAChRs are decreased but there are no significant changes to the expression level of the M_1_ mAChRs when compared to normal aged brain tissue (Nordberg and Winblad, 1986; Flynn et al., 1991; Hampel et al., 2018); an important consideration for therapeutic development. Treatment for Alzheimer's disease is currently achieved with the use of acetylcholinesterase inhibitors, which prevent the degradation of ACh in the synapse, thus directly increasing cholinergic signaling (Lleo, 2007). Unfortunately, this treatment is only modestly effective for treating the cognitive symptoms and does not treat the underlying pathology of the disease itself, only delaying the symptomatic progression of the disease. Acetylcholinesterase inhibitors are associated with dose-limiting gastrointestinal side effects, including nausea, vomiting and diarrhea, due to the global increase in ACh throughout the periphery (Lockhart et al., 2009; Harvey, 2012). As an alternative to increasing ACh levels, directly targeting the mAChRs with agonist ligands could increase neuronal stimulation to improve cognitive functions. This hypothesis was indeed tested in human clinical trials with the rigid ACh analogue, AF102B (cevimeline) or with the M_1_/M_4_ mAChR-preferring agonist, xanomeline (Fisher et al., 1996; Bodick et al., 1997). Treatment with either cevimeline or xanomeline significantly improved the cognitive abilities of Alzheimer's disease patients when compared with placebo controls (Fisher et al., 1996; Bodick et al., 1997), proving that direct activation of the mAChRs is a feasible drug targeting strategy. However, like the acetylcholinesterase inhibitors, cevimeline and xanomeline both display similar gastrointestinal side effects and can cause syncope in patients (Fisher et al., 1996; Bodick et al., 1997). Although directly targeting the mAChRs remains a possible approach for developing new therapies for cognitive enhancement, the use of mAChR agonists is currently limited by the side effects mediated by activation of peripherally expressed mAChRs.

#### Amyloid Beta Processing by the M_1_ mAChR

Cleavage of the amyloid precursor protein (APP) by secretase enzymes produces several amyloid-β peptides (Kar et al., 2004). Disturbances in APP processing increase the production of toxic amyloid-β proteins that form plaques on neurons, disrupt neurotransmission and cause neuronal cell death (Harkany et al., 1995; Kelly et al., 1996; Kar et al., 2004; Hartl et al., 2015). Interestingly, these amyloid-β proteins directly affect the coupling of the M_1_ mAChR to G proteins (Janickova et al., 2013). In [^3^H]-N-methyl-scopolamine radioligand binding experiments, high (G protein-coupled state) and low (G protein-uncoupled state) affinity binding sites can be defined by increasing concentrations of the agonist, carbachol in M_1_ mAChR expressing Chinese hamster ovary (CHO) cells. Following a four day pre-treatment of the cells with amyloid-β(1-42), the high affinity binding site is lost, suggesting that the peptide disrupts G protein coupling to the M_1_ mAChR (Janickova et al., 2013). Carbachol-stimulated GTPase activity, IP_3_ production and intracellular calcium release are also inhibited in M_1_ mAChR expressing CHO cells and rat neocortical cultures following a 24–96 h pre-treatment with amyloid-β(1-40/42) (Kelly et al., 1996; Janickova et al., 2013). Thus the amyloid-β peptides can uncouple the M_1_ mAChR from G proteins and affect Gα_q_-mediated signaling events. This is an important point to consider for the development of M_1_ mAChR selective ligands for the treatment of Alzheimer's disease. In M_1_ mAChR knockout mice, amyloid-β peptide production is increased, while reintroduction of the M_1_ mAChR into neuronal cultures from these mice reverses the changes in amyloid-β peptide production (Davis et al., 2010a). In addition, activation of the M_1_ mAChR with agonists increases the production of soluble APP and decreases the production of amyloid-β, suggesting that direct activation of this receptor may also yield disease modifying benefits (Fisher et al., 2003).

### Schizophrenia

Dopamine abnormalities consisting of low dopamine levels in the cortex and high dopamine levels in the striatum are classically proposed to cause the main symptoms of schizophrenia (Weinberger, 1987; Kesby et al., 2018). Positron emission tomography imaging shows that people with schizophrenia have increased synaptic dopamine levels, release higher levels of dopamine in response to amphetamines and have increased basal dopamine synthesis capacity (Kesby et al., 2018). The altered dopamine levels contribute to three distinct symptom domains: positive symptoms (delusions, hallucinations), negative symptoms (lack of emotional expression, low motivation and social withdrawal) and cognitive symptoms (impaired learning, memory, attention and executive function) (Kesby et al., 2018). Interestingly, activation of the M_4_ mAChR in the striatum controls the release of dopamine through a mechanism involving 2-arachidonoylglycerol and glutamate release, acting upon the cannabinoid receptors (CB2) and the glutamate receptors (mGluR1), respectively (Foster et al., 2016; Yohn et al., 2018). In striatal projection neurons, the M_4_ mAChR is highly co-expressed with the D_1_ dopamine receptor (Jeon et al., 2010). When oxotremorine, a mAChR agonist, is administered to these neurons, decreases in dopamine-stimulated cAMP signaling are observed (Nair et al., 2019). This occurs via crosstalk between the receptors, where dopamine activates cAMP signaling via the Gα_s_-coupled D_1_ dopamine receptor, and ACh inhibits cAMP production via the Gα_i/o_-coupled M_4_ mAChR. This complex interplay between the different neurotransmitter systems highlights how modulation of one system can perturb the network of neurotransmission in the brain.

Studies using post-mortem brain tissues indicate that there is a reduction in the expression levels of both the M_1_ and the M_4_ mAChRs in the caudate, putamen, hippocampus, cingulate cortex and the prefrontal cortex in schizophrenic brains compared to non-schizophrenic brains (Raedler et al., 2007; Dean et al., 2016). This suggests that a decrease in signaling from both of these receptors may contribute to symptoms of schizophrenia. A role for the M_4_ mAChR is further supported by evidence from human clinical trials, where the M_1_/M_4_-preferring agonist, xanomeline, improved the positive, negative and cognitive symptoms in schizophrenic patients (Shekhar et al., 2008). While these results are encouraging, an unfavourable, peripheral side effect profile has prevented xanomeline progressing further into the clinic. In 2018, Karuna therapeutics created ‘KarXT’, a combination therapy of xanomeline and trospium (a peripherally restricted mAChR antagonist) (Brannan et al., 2020). In KarXT, trospium specifically blocks the peripheral actions of xanomeline, while allowing xanomeline to provide therapeutic efficacy in the CNS. In phase IIb clinical trials, KarXT successfully reduced positive, negative and cognitive symptoms in schizophrenic patients, but some anti-muscarinic side effects such as constipation, nausea, dry mouth, dyspepsia and vomiting were still observed (Brannan et al., 2020). While these clinical results are very promising, they also suggest that further improvements could be made to reduce the adverse effects of mAChR targeting treatments.

#### Muscarinic Receptor-Deficit Schizophrenia

Schizophrenia is a syndrome that is likely composed of multiple etiologies, but presents with similar symptoms. Thus, in the path forward for treatments, the syndrome should be broken up into distinct biological problems that can be solved with the correctly tailored treatment (Jablensky, 2006). [^3^H]-pirenzipine binding in human post-mortem brain slices identified a subset (∼25%) of schizophrenic patients that have a reduction of approximately 75% M_1_ mAChR expression in the Brodmann's area nine of the pre-frontal cortex when compared to non-schizophrenic controls (Salah-Uddin et al., 2009; Scarr et al., 2009; Dean et al., 2016; Scarr et al., 2018). Patients with this reduced M_1_ mAChR expression profile have been classified as a distinct subset of patients with ‘muscarinic receptor-deficit syndrome’ (MRDS) schizophrenia. Gene expression microarray data indicate that 65 genes are distinctly altered in the MRDS group; these genes are important for controlling cell movement and cell signaling pathways, upstream of M_1_ mAChR (Scarr et al., 2018). This finding is important, because treatment of this subset of schizophrenic patients with M_1_ mAChR-selective ligands may fail. Indeed, in [^3^H]-N-methyl-scopolamine binding experiments using post-mortem brain tissues from MRDS and non-schizophrenics, an M_1_ mAChR-selective ligand, benzyl quinolone carboxylic acid (BQCA), had much weaker effects in the MRDS tissues than in the non-schizophrenic control tissues (Dean et al., 2016; Hopper et al., 2019). Thus, targeting an alternative receptor, such as the M_4_ mAChR, may be more beneficial for this subgroup of schizophrenic patients.

## Understanding the Role of the M_1_ and M_4_ mAChRs Through the Use of Genetically Modified Mice

Important information about the role that the M_1_ and M_4_ mAChRs play in the brain can be gained through the use of receptor knockout and chemogenetically modified receptor mice. Knockout of the gene of interest often results in biochemical and behavioral changes that can then be associated with the function of that particular gene. An alternative mouse model uses a mutant mAChR that has two orthosteric binding site mutations, which cause the endogenous ligand ACh to lose activity at the receptor. This mutant receptor, known as a "Designer Receptor Exclusively Activated by Designer Drugs" (DREADD) can however, be activated by the otherwise pharmacologically inactive compound, clozapine-N-oxide (Roth, 2016).

### M_1_ and M_4_ mAChR Knockout Mice Are Hyperactive

Knockout of either M_1_ or M_4_ mAChRs produces hyperactive mice relative to wild-type controls, suggesting that both mAChRs play a role in the control of locomotor activity (Gomeza et al., 1999; Miyakawa et al., 2001; Wess, 2004; Koshimizu et al., 2012). It should be noted, however, that the hyperactivity effect is greater in the M_1_ knockout mouse than in the M_4_ knockout mouse (Wess, 2004). The M_1_ DREADD mice behave similarly to the M_1_ knockout mice, with respect to locomotor activity levels; however, the hyper-locomotor activity is reversed when the M_1_ DREADD is activated following administration of clozapine-N-oxide (Bradley et al., 2020). This hyperactivity may be linked to increases in dopamine release, since increases in striatal extracellular dopamine were observed by *in vivo* quantitative microdialysis in the M_1_ mAChR knockout mice (Gerber et al., 2001; Zhang et al., 2002). In a striatal specific M_4_ mAChR knockout, dopamine efflux increases in the nucleus accumbens, which increases cAMP signaling through the D_1_ dopamine receptor (Jeon et al., 2010). These results suggest that both M_1_ and M_4_ mAChR subtypes play a role in the control of central dopamine signaling, which can be linked to increased locomotor activity in mice.

Dopamine transporter knockout mice have a reduction in dopamine reuptake from the synapse, resulting in an accumulation of synaptic dopamine levels and a characteristic hyperactivity phenotype (Gainetdinov et al., 1999; Carpenter et al., 2012; Nair et al., 2019). Thus, measurement of locomotor activity is a commonly used behavioral assay to assess antipsychotic medications. Ligand-induced hyperactivity, using amphetamine, MK-801 or phencyclidine, is typically reduced by compounds that are efficient antipsychotics. Muscarinic agonists acting at either the M_1_ or the M_4_ mAChRs decrease the level of synaptic dopamine; therefore, increasing cholinergic activity at both the M_1_ and M_4_ mAChR subtypes may be helpful in treating the psychotic symptoms of schizophrenia. Intriguingly, M_1_ mAChR-mediated locomotor activity is linked to Gα_q_-mediated signaling events; in mice that express a phosphodeficient M_1_ mAChR (G protein-signalling pathway biased) the locomotor activity was equivalent to the wild type mice when monitored over a 24 h period (Bradley et al., 2020). In addition, monitoring another Gα_q_-linked signaling event, ligand-induced IP_1_ accumulation in the striatum, can predict the behavioral effects of ligands toward reversing amphetamine-induced locomotor activity (Popiolek et al., 2016). Thus M_1_ mAChR-selective compounds that activate Gα_q_-IP_3_ pathways may exhibit good antipsychotic efficacy.

### M_1_ and M_4_ mAChR Knockout Mice Are Anxious

Initial characterisation of M_1_ mAChR knockout mice in the elevated plus maze, a mouse model of anxiety, indicated that the M_1_ mAChR knockout mice and wild type mice both spent a similar amount of time in the open arms, suggesting that M_1_ mAChR knockout is not associated with anxiety (Miyakawa et al., 2001). However, in more recent studies using the same behavioral paradigm, M_1_ mAChR knockout, M_1_ DREADD and M_1_ mAChR-phosphodeficient mice spent less time exploring the open arms than the wild-type controls (Bradley et al., 2020). This latter study suggests that the loss of the M_1_ mAChR does indeed produce mice with greater anxiety. Interestingly, the amount of time the M_1_ DREADD mice spent exploring the open arms was restored to that of the control mice upon administration of clozapine-N-oxide, further supporting a role for the M_1_ mAChR in anxiety (Bradley et al., 2020). The M_1_ mAChR driven anxiety was also apparent in the M_1_ mAChR-phosphodeficient mouse, suggesting that the anxiety occurs downstream of G protein-dependent signaling pathways for the M_1_ mAChR (Bradley et al., 2020). The only study that explored anxiety responses in M_4_ mAChR knockout mice to date revealed that M_4_ mAChR knockout mice have decreased burying responses when tested in a shock-probe burying model, indicating that they also have increased anxiety levels (Degroot and Nomikos, 2006).

### M_4_ mAChR Knockout Mice Have Impaired Social Interactions

Knockout of the M_4_ mAChR generates mice with only subtle physiological changes when they are compared to wild type mice, indicating that this is a very mild receptor knockout (Gomeza et al., 1999; Bymaster et al., 2001; Bymaster et al., 2003; Koshimizu et al., 2012). M_4_ mAChR knockout mice display abnormal social behavior, with less contact observed when compared to wild type mice (Koshimizu et al., 2012). This contrasts with the results in the M_1_ mAChR knockout mice, where increases in social contacts are observed compared to wild type mice, which may be due to the increase in locomotor activity of these mice rather than a reduction in anxiety (Miyakawa et al., 2001). The M_4_ mAChR may be involved in psychotic symptoms because the M_4_ mAChR knockout mice have increased sensitivity to psychomimetic drugs, with increased startle responses observed when M_4_ mAChR knockout mice are given phencyclidine (an NMDA receptor antagonist) compared with wild type controls (Felder et al., 2001; Bubser et al., 2014). The pre-pulse inhibition of the startle reflex behavioral paradigm is another test used to assess antipsychotic drugs for efficacy. When the M_4_ mAChR knockout mice are pre-treated with phencyclidine, these animals have increased disruption of pre-pulse inhibition, suggesting that the M_4_ mAChR may be involved in psychosis (Felder et al., 2001; Wess 2004). When M_4_ mAChR knockout mice were tested in the Morris water maze or by touchscreen discrimination tasks, they performed equally well as wild type controls, suggesting that the M_4_ mAChR has little involvement in cognition (Koshimizu et al., 2012; Bubser et al., 2014). Together these results suggest that the M_4_ mAChR is important in psychosis, social behavior and anxiety but is less important in cognition. Thus, M_4_ mAChR-targeting drugs may be particularly helpful for treating the positive and negative symptoms of schizophrenia.

## Targeting Specific mAChR Subtypes

### Multiple Binding Sites at mAChRs

**FIGURE 2:**
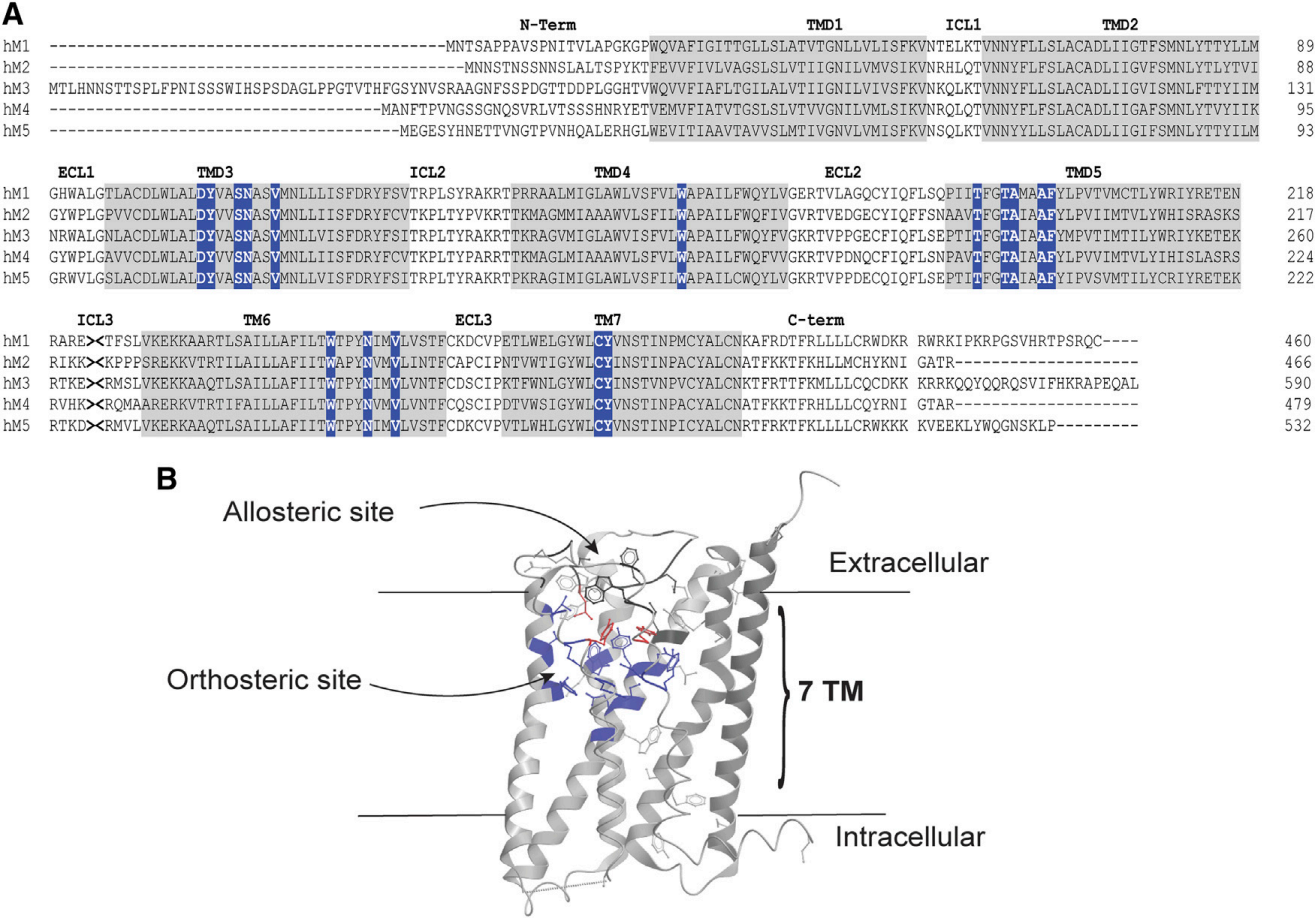
Orthosteric and allosteric sites of the mAChRs. **(A)** Amino acid sequence alignment of the human M_1_-M_5_ mAChRs. The amino (N)-terminal tail, Intracellular loops (ICL), extracellular loops (ECL) and carboxy (C)-terminal tails are shown as black letters on a white background. Transmembrane domains (TMD) are shown as black letters on a gray background. Orthosteric site residues are white letters on a blue background. The intracellular loop three was truncated, as indicated by >< for presentation of the alignment. Alignment was performed using clustal omega. **(B)** X-ray crystal structure of the human M_4_ mAChR (RCSB PDB number 5DSG), showing the location of orthosteric and allosteric sites. Orthosteric site residues are highlighted in blue, allosteric site residues are highlighted in black and residues that contribute to both binding pockets are highlighted in red.

Historically, it has proven extremely challenging to selectively target one mAChR subtype over the other mAChR subtypes because the residues lining the orthosteric ACh binding site of all five mAChR subtypes are absolutely conserved. Figure 2 shows the alignment for the amino acid residues of the M_1_-M_5_ mAChRs. This absolute conservation of the residues within the orthosteric site has thus hindered the design and synthesis of highly subtype selective mAChR ligands. It is thought that activation of the peripheral mAChRs by non-selective orthosteric ligands is one of the main reasons for the failure of many mAChR-based drug candidates for the treatment of neurological disorders.

The orthosteric site lies deep within the transmembrane helices of the mAChRs, defined by amino acid residues in the transmembrane spanning helices 3, 4, 5, 6, and 7, as shown in Figure 2B (Thal et al., 2016). Interestingly, it appears that all mAChRs have an allosteric binding site that is located in an extracellular vestibule above the orthosteric site (Haga et al., 2012; Kruse et al., 2013; Thal et al., 2016). Allosteric sites recognize structurally distinct ligands to modulate the activity of co-bound orthosteric ligands (Christopoulos and Mitchelson, 1995; Christopoulos et al., 1997; Lanzafame et al., 1997; Christopoulos et al., 1998). Gallamine was one of the earliest and best-studied allosteric ligands for the mAChRs, and remains an exemplar molecule for GPCR allostery in general (Clark and Mitchelson, 1976). Through receptor mutagenesis and structural biology studies, many residues that are important for binding allosteric ligands into the allosteric site of the M_1_ and M_4_ mAChRs have been identified in this region (Nawaratne et al., 2010; Leach et al., 2011; Abdul-Ridha et al., 2014; Keov et al., 2014; Thal et al., 2016). The important residues for this site include those in the top of transmembrane helices 2, 6, and 7 as well as in extracellular loop 2 (Nawaratne et al., 2010; Leach et al., 2011; Abdul-Ridha et al., 2014; Keov et al., 2014; Thal et al., 2016). Excitingly, the amino acid residues of this extracellular allosteric site of the mAChRs show greater diversity between the different subtypes, thus providing the framework for designing mAChR subtype selective allosteric ligands. In fact, the allosteric sites of the M_1_ and M_4_ mAChRs have successfully been targeted by rationally designed synthetic allosteric ligands, with (now) a large number of subtype selective allosteric ligands available as pharmacological tools (Ma et al., 2009; Kuduk et al., 2010; Kuduk et al., 2011; Salovich et al., 2012; Le et al., 2013; Mistry et al., 2013; Croy et al., 2014; Huynh et al., 2015; Davoren et al., 2016a; Mistry et al., 2016a; Wood et al., 2016a; Davoren et al., 2016b; Mistry et al., 2016b; Wood et al., 2016b; Wood et al., 2017a; Wood et al., 2017b; Davoren et al., 2017; Long et al., 2017; Tarr et al., 2017; Bertron et al., 2018; Beshore et al., 2018; Dallagnol et al., 2018; Engers et al., 2019a; Engers et al., 2019b; Chopko et al., 2019; Jorg et al., 2019; Poslusney et al., 2019; Schubert et al., 2019; Temple et al., 2019; Temple et al., 2020a; Temple et al., 2020b). Since the orthosteric and allosteric sites are topographically distinct, two ligands can bind one receptor simultaneously. Upon binding to the receptor, the allosteric ligand can alter the pharmacological properties of the co-bound orthosteric ligand. This alteration in the pharmacological profile of the orthosteric ligand is defined as an ‘allosteric interaction’ (Christopoulos et al., 2014), and can be identified as either a change of binding affinity (K_A_) or signaling efficacy (τ_A_) of the orthosteric ligand at the receptor.

### Types of Allosteric Modulators

Several different types of allosteric modulators have been identified thus far, depending on their effect on the co-bound orthosteric ligand and their potential direct effect on receptor-mediated signaling. Positive allosteric modulators (PAMs) are ligands that enhance the pharmacological properties of the orthosteric ligand. Negative allosteric modulators (NAMs) are ligands that diminish the affinity and/or efficacy of the orthosteric ligand. Neutral allosteric ligands (NALs) are ligands that bind to the allosteric site, but do not alter the properties of the co-bound orthosteric ligand (May et al., 2007; Christopoulos et al., 2014). In addition to their potential modulatory effects on orthosteric ligand binding and/or signaling, allosteric ligands can also have a direct effect on the receptor, and trigger a signaling response, in a not too dissimilar manner to orthosteric agonists, but from the allosteric site specifically. These ligands are called ‘PAM-agonists’ as they potentiate the effects of the orthosteric ligand and simultaneously have a direct effect on the receptor. This is in contrast to “pure” PAMs, which only potentiate the effect of the orthosteric ligand but have no direct effect on the receptor’s signaling capabilities on their own. The discovery of allosteric modulators of mAChRs has rejuvenated drug discovery programs, due to their significant advantages over orthosteric drugs. They can achieve subtype selectivity, maintain normal spatial and temporal profiles of physiological signaling and can be devoid of agonist activity, a key feature for targeting mAChRs in the CNS.

### Quantifying Allosteric Interactions

**FIGURE 3:**
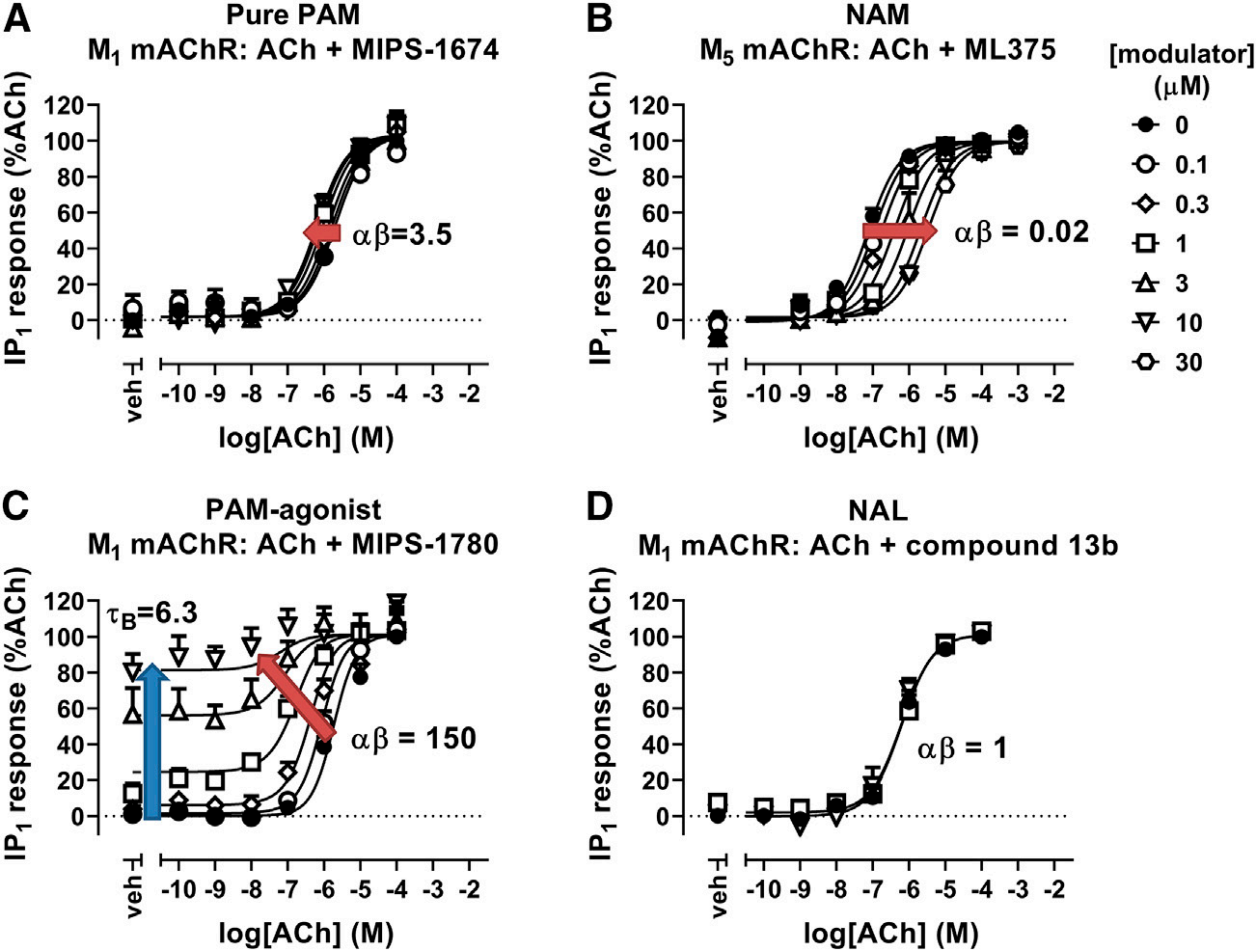
Allosteric modulation of mAChRs. **(A)** The PAM, MIPS-1674, increases the potency (αβ) of ACh toward IP_1_ accumulation at the M_1_ mAChR. **(B)** The NAM, ML375, decreases the potency of ACh at the M_5_ mAChR in an IP_1_ accumulation assay. **(C)** The PAM-agonist, MIPS-1780, increases the potency (αβ) of ACh toward IP_1_ accumulation, but also has its own allosteric agonist effect (τ_B_). **(D)** The NAL, compound 13b, has no effect on the potency of ACh toward IP_1_ accumulation at the M_1_ mAChR. Data are from van der Westhuizen et al. (2018), Berrizzi et al. (2016), and Jorg et al. (2020). Data were fit with the simplified operational model of allostery and agonism (Aurelio et al., 2009), to quantify the cooperativity (αβ) and intrinsic efficacy (τ_B_) parameters.

There are a variety of biochemical and functional approaches for experimentally determining and validating allosteric interactions (Christopoulos, 2014; Christopoulos et al., 2014). The majority involve the determination of the effect of increasing concentrations of an allosteric modulator on the binding or signaling of an orthosteric ligand. In the case of a PAM, the allosteric effect may result in an increase in orthosteric ligand affinity, which would manifest as an increase in the dissociation constant (K_A_) and the functional potency (EC_50_) of the orthosteric ligand, and/or an increase in ligand efficacy, which would manifest functionally as an increase in agonist potency for full agonists or an increase in agonist maximal effect (E_max_) for partial agonists. In contrast, a NAM would have the opposite effects on orthosteric ligand affinity and/or efficacy, thus reducing agonist affinity, potency and/or E_max_ depending on the type of NAM effect and the intrinsic efficacy of the orthosteric ligand (Figure 3). If the allosteric modulator is a NAL, then there will be no change in the affinity or potency of the orthosteric ligand at equilibrium, although the NAL may still result in changes on orthosteric agonist binding kinetics as well as competing with PAMs or NAMs that bind to the same allosteric site (Christopoulos et al., 2014). Additional effects can be observed, depending on the properties of the allosteric modulator. For example, if the allosteric ligand is a PAM-agonist, it can activate the receptor in the absence of an orthosteric ligand in addition to allosteric modulatory effects (Figure 3).

To quantify the effects that an allosteric modulator can exert on an orthosteric ligand, a number of mechanistic and operational models have been developed for analysis of experimental data. The most common mechanistic model that describes allosteric effects on orthosteric ligand affinity is the allosteric ternary complex model (ATCM; Stockton et al., 1982; Ehlert, 1988; Christopoulos and Kenakin, 2002). The ATCM quantifies the affinity of the allosteric modulator for the allosteric site (K_B_) and the effect the modulator has on the affinity of the co-bound orthosteric ligand (binding cooperativity; α), in addition to orthosteric ligand affinity (K_A_). Values of α > 1 denote positive cooperativity, values of *α* < 1 but > 0 denote negative cooperativity, and *α* values equal to one denote neutral binding cooperativity. For signaling assays, the ATCM has been incorporated into an operational model that also allows for quantification of allosteric effects on orthosteric agonist efficacy (*β*) as well as direct orthosteric (τ_A_) and allosteric (τ_B_) agonism (Price et al., 2005; Leach et al., 2007). In operational terms, *β* values of >1 denote positive efficacy modulation, values of *β* < 1 but > 0 denote negative efficacy modulation, and *β* values equal to one denote no modulation of orthosteric signaling efficacy. Of note, in situations where the orthosteric ligand is a full agonist in both the absence and presence of modulator, the operational model can be simplified to allow for derivation of an overall combined modulatory effect, quantified through the composite parameter, αβ (Aurelio et al., 2009). Application of these allosteric models has been successful in determining structure activity relationships for allosteric modulators of multiple GPCRs including the adenosine A_1_ (Ferguson et al., 2008; Aurelio et al., 2009; Aurelio et al., 2010; Aurelio et al., 2011; Valant et al., 2012a) the mAChRs (Mistry et al., 2013; Mistry et al., 2016b; Dallagnol et al., 2018; Jorg et al., 2019; Jorg et al., 2020), the mGlu receptors (Mueller et al., 2012; Gregory et al., 2013; Turlington et al., 2013) and the GLP1-R (Wootten et al., 2012; Wootten et al., 2013b; Hager et al., 2017).

### M_1_ and M_4_ mAChR-Selective Positive Allosteric Modulators

The first M_1_ mAChR PAM with high subtype-selectivity was presented by Merck with the discovery of BQCA (Ma et al., 2009). BQCA increases the binding affinity and functional potency of ACh and carbachol at the M_1_ mAChR overexpressed in CHO cells or primary cortical neurons (Ma et al., 2009; Canals et al., 2012; Abdul-Ridha et al., 2013). *In vivo* animal studies also showed that BQCA reverses scopolamine-induced memory loss, decreases amphetamine-induced hyperlocomotion in rodents (Ma et al., 2009; Chambon et al., 2012) and can act synergistically with sub-threshold doses of antipsychotics (Choy et al., 2016). Notably, it also increases APP processing (Shirey et al., 2009) and extends the lifespan of terminally-sick mice with neurodegenerative disease (Bradley et al., 2017), suggesting that M_1_ PAMs have the potential to improve cognition and to modify the underlying cause of Alzheimer's disease. However, this compound was not progressed into clinical trials due to its poor solubility, limited brain penetration and high plasma protein binding properties (Kuduk et al., 2011). Subsequently, there have been substantial efforts to develop novel M_1_ mAChR PAMs with improved physicochemical properties (Mistry et al., 2013; Davie et al., 2014; Kuduk et al., 2014; Davoren et al., 2016a; Mistry et al., 2016a; Davoren et al., 2016b; Mistry et al., 2016b; Panarese et al., 2016; Davoren et al., 2017; Flohr et al., 2017; Bertron et al., 2018; Beshore et al., 2018; Dallagnol et al., 2018; Engers et al., 2019b; Jorg et al., 2019; Mandai et al., 2019; Jorg et al., 2020)

High M_4_ mAChR-subtype selectivity was first described for the PAM, LY2033298 (Chan et al., 2008). This PAM increased the binding affinity and potency of ACh in CHO cells expressing the human M_4_ mAChR, however, LY2033298 was also noted to have some activity at the M_2_ mAChR (Chan et al., 2008; Valant et al., 2012b). In addition, LY2033298 did not potentiate ACh at the rodent M_4_ mAChR to the same extent as at the human M_4_ mAChR, indicating that there is species variability in the modulatory responses at the M_4_ mAChR (Chan et al., 2008; Suratman et al., 2011). When administered *in vivo*, LY2033298 has weak to modest effects. However, when LY2033298 is co-administered with a sub-effective dose of oxotremorine, it reduced conditioned avoidance responses and reversed apo-morphine induced pre-pulse inhibition (Chan et al., 2008; Leach et al., 2010). This compound provided important proof of concept that the M_4_ mAChR can be allosterically targeted and has served as a basis for the development of novel compounds through medicinal chemistry efforts (Salovich et al., 2012; Huynh et al., 2013; Le et al., 2013; Byun et al., 2014; Huynh et al., 2015; Wood et al., 2016a; Wood et al., 2016b; Wood et al., 2017a; Bewley et al., 2017; Wood et al., 2017b; Tarr et al., 2017; Engers et al., 2019a; Schubert et al., 2019; Temple et al., 2019; Temple et al., 2020a; Temple et al., 2020b).

Achieving receptor subtype selectivity was an important milestone in the development of potentially therapeutic mAChR ligands. Unfortunately, most subtype-selective PAMs that have been disclosed to date are still plagued by both central and peripheral adverse side effects, particularly at the M_1_ mAChR. One of the most worrying CNS-based side effects of M_1_ mAChR-selective compounds is their ability to trigger epileptic like seizures (Turski et al., 1989; Davoren et al., 2016a; Bradley et al., 2020). M_1_ mAChR-selective PAMs, such as PF06767832 and MK-7622, have high intrinsic agonism toward calcium signaling in transfected cell systems, and trigger seizures in rodents. It is hypothesised that this on-target effect is due to the strong allosteric agonism (τ_B_) of these ligands (Davoren et al., 2016a; Beshore et al., 2018; Moran et al., 2019). This adverse effect is not observed in M_1_ mAChR knockout mice administered orthosteric agonists, confirming that the seizure events are indeed driven by the M_1_ mAChR (Hamilton et al., 1997). Therefore, focusing the development of next generation selective PAMs on compounds with minimal intrinsic allosteric efficacy and/or modest positive cooperativity may eliminate this and other on-target side effects while retaining the therapeutic benefit of allosteric potentiation of the ACh response.

## Subtype-Selective Biased Allosteric Modulators to Improve Therapeutic Efficacy

### Biased Signaling

GPCRs are highly dynamic cell-surface proteins that can activate multiple signaling pathways through recruitment and activation of different families of G proteins, cellular kinases and scaffold proteins. Excitingly, there is a large body of evidence suggesting that recruitment and activation of the different transducer proteins can occur in a ligand-dependent and in a cell-type-dependent manner (Smith et al., 2018; Wootten et al., 2018). It is now clear that structurally distinct ligands can stabilise different receptor conformations. Whilst some GPCR ligands are capable of stabilising a large set of receptor conformations, allowing the receptor to couple to the full range of transducers to transmit the signals from the extracellular environment to intracellular proteins, others can only stabilise a subset of conformations. Such ligands are called biased ligands, because they can direct the signal that emanates from the receptor to one or several particular signaling pathways over all the signaling pathways that are available to the GPCR (Smith et al., 2018; Wootten et al., 2018). This ability to selectively activate certain pathways at the detriment of others has made the concept of bias agonism extremely attractive for development of drugs targeting the M_1_ and M_4_ mAChRs. If some of the side effects observed with mAChR agonists and allosteric modulators are due to on-target driven side effects, then developing selective biased agonists or modulators for these receptors may be another method to reduce the adverse effects of drugs.

Biased agonists often display a reversal of efficacy and/or potency in concentration-response curves at different signaling pathways relative to a reference compound. Such effects have been well documented for many GPCRs (Smith et al., 2018; Wootten et al., 2018). Relative agonist activity values are then calculated for a range of different ligands and for a range of different pathways then compared to a reference agonist that ideally activates all known coupled pathways. There are several methods that have been proposed to quantify bias at GPCRs (Ehlert, 2008; Rajagopal et al., 2011; Kenakin, 2012; Kenakin et al., 2012; Kenakin and Christopoulos, 2013). These models essentially condense comparisons between a test and reference ligand's affinity, potency and efficacy into a single parameter, which is then used to compare the effects of ligands on different pathways relative to the reference agonist. Due to the difficulties in achieving subtype selectivity with orthosteric agonists at the mAChRs, engendering biased allosteric modulation is a viable strategy for achieving subtype-selective biased signaling at mAChRs. Allosterically-mediated bias can be experimentally observed by changes in the efficacy parameter (*β*), where an allosteric modulator can impose positive modulation of an orthosteric agonist toward one pathway, but may yield negative modulation of the same co-bound orthosteric agonist on a distinct signaling pathway (Goupil et al., 2010; Wootten et al., 2013a). More subtly, it can also manifest as a variation in the cooperativity that an allosteric ligand may exert on an orthosteric ligand, or as a change in the functional affinity (K_B_) of allosteric ligands for cellularly-compartmentalised receptor conformations linked to different signaling pathways (Davey et al., 2012; Wootten et al., 2013a).

Excitingly, the physiological relevance of biased signaling was recently shown using phospho-deficient and chemogenetically modified M_1_ mAChRs mouse models (Bradley et al., 2020). This study suggested that mAChR ligands that induce M_1_ mAChR phosphorylation are critical for reducing adverse effects, such as salivation and gastrointestinal disturbances. In contrast, mAChR ligands that displayed weaker ability to phosphorylate M_1_ mAChR, thus biased toward G protein signaling pathways, produced greater central and peripheral adverse effects (Bradley et al., 2020). When all the phosphorylation sites of the M_1_ mAChR were mutated to alanine to create an M_1_-phophodeficient mAChR, activation of Gα_q_-mediated signaling events remained intact, but recruitment of β-arrestin was decreased by ∼50% (Bradley et al., 2020). Excitingly, M_1_-phosphodeficient mAChR transgenic mice exhibited similar locomotor activity over 24 h compared to wild type mice, but displayed increased anxiety-like behaviors and cognitive deficits in a Y-maze test, similar to M_1_ mAChR knockout mice (Bradley et al., 2020). Together, these ground-breaking studies suggest that the regulation of learning and memory processes and anxiety-related behaviors, are directly linked to M_1_ mAChR phosphorylation and subsequent downstream signaling pathways, such as β-arrestin recruitment, whereas, adverse effects such as salivation, seizures, and hyperactivity are linked to Gα_q_, IP_3_ and calcium signaling pathways (Bradley et al., 2020). M_1_ mAChR-selective positive allosteric modulators that stabilize mAChR conformations linked to receptor phosphorylation and β-arrestin signaling pathways may be strong drug candidates to improve cognition in Alzheimer's disease. In contrast, given that the locomotor activity is linked to G protein signaling events, drugs that exhibit strong phosphorylation and β-arrestin signaling pathways, yet retain some degree of signaling via G proteins may be superior for treating some symptom domains of schizophrenia.

### Biased Ligands for the M_1_ and M_4_ mAChRs

#### Biased Orthosteric Agonists

**FIGURE 4:**
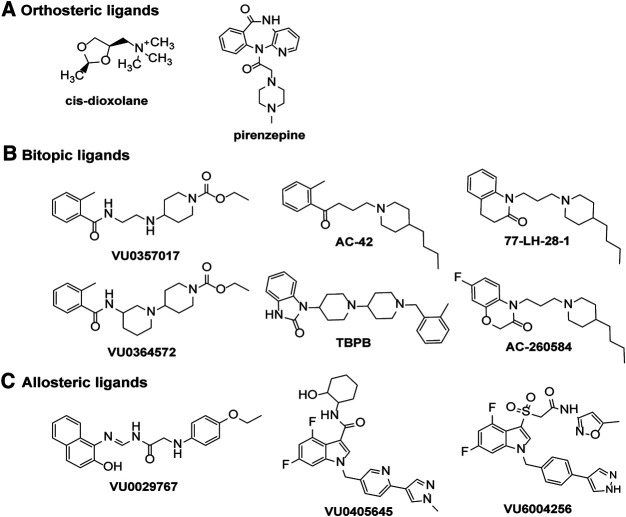
Chemical structures of ligands displaying signaling bias at the M_1_ or M_4_ mAChRs. Biased signaling profiles are reported for **(A)** orthosteric agonists or antagonists, **(B)** bitopic ligands or **(C)** allosteric ligands at the M_1_ and M_4_ mAChRs.

Although there are a wide range of ligands available for the mAChRs, only a handful of studies have systematically tested for biased agonists and allosteric modulators at the M_1_ and M_4_ mAChRs. The M_1_ mAChR couples to Gα_q/11_, Gα_i/o_ and Gα_s_ proteins, to increase calcium release, inhibit and stimulate cAMP, respectively. Early studies that characterised mAChR agonist profiles across different receptor subtypes and signaling pathways suggested that pilocarpine, oxotremorine, arecoline, cevimeline, McN-A-343 and *cis*-AF30, were Gα_q_-biased ligands that did not activate Gα_s_ and cAMP signaling at the M_1_ mAChR in CHO cells (Table 1; Gurwitz et al., 1994). However, subsequent studies have demonstrated that pilocarpine and arecoline are weak partial agonists for the cAMP signaling pathway (Thomas et al., 2008). Interestingly, the non-selective agonist, *cis*-dioxolane, increases arachidonic acid release and cAMP accumulation without activating IP_3_ signaling (Figure 4, Table 1) (Gurwitz et al., 1994), a finding that has not been confirmed by other studies but could represent a potentially interesting bias profile. At the M_1_ mAChR expressed in CHO cells, five agonists were investigated for biased signaling toward Gα_q_ activation, Gα_i__1/2_ activation, Gα_s_ activation, IP_3_ accumulation and cAMP accumulation (Table 1) (Thomas et al., 2008). The non-subtype selective mAChR agonists, oxotremorine-M, pilocarpine and arecoline activated Gα_q_, Gα_i__1/2_, IP_3_ and cAMP pathways. Whereas, the M_1_-selective bitopic ligands, AC-42 and 77-LH-28–1 activated Gα_q_, IP_3_ and cAMP pathways but did not activate Gα_i__1/2_, suggesting that the allosteric ligands were biased toward Gα_s_ and Gα_q_-coupled pathways (Figure 4, Table 1) (Thomas et al., 2008; Davis et al., 2009).

**TABLE 1:** Ligands screened, pharmacology assessed and biased profiles determined for a range of orthosteric, bitopic and allosteric ligands at the M_1_ mAChR. Different combinations of ligands and pathways have been used to assess a range of mAChR ligands for biased signaling. Biased signaling has been assessed in recombinant Chinese hamster ovary (CHO) or Human embryonic kidney (HEK) cells stably expressing either the rat (rM_1_) or human (hM_1_) mAChRs, OC-033 mouse tumor cell lines or Sprague-Dawley rat dorsal root ganglia (DRG) neurons.

Ligand	Pharmacology	Bias profile	References
Acetylcholine	Agonist. Efficacy on IP_3_, AA, ↑cAMP, pERK1/2, β-arrestin pathways. (CHO-hM_1_ or rM_1_ cells)	Non-biased	Gurwitz et al. (1994), Keov et al., 2014, and van der Westhuizen et al. (2018)
Cabachol	Agonist. Efficacy on IP, Ca^2+^, AA, ↑cAMP, β-arrestin pathways. (CHO-hM_1_ or rM_1_; HEK-hM_1_ OC-033-rM_1_, HEK-CRISPR/Cas9-Δβ-arrestin, rat DRG cells cells)	Non-biased	Gurwitz et al. (1994), Davis et al. (2009), Davis et al. (2010a), and Sabbir and Fernyhough (2018)
Oxotremorine-M	Agonist. Efficacy on G_q_, IP_3_, AA, ↑cAMP, G_i/o_, β-arrestin pathways. (CHO-hM_1_ or rM_1_ cells)	Non-biased	Gurwitz et al. (1994), Thomas et al. (2008), Davis et al. (2009), and Davis et al. (2010b)
Oxotremorine	Agonist. Efficacy on IP_3_, AA, ↑cAMP pathways. (CHO-rM_1_ cells)	Non-biased	Gurwitz et al. (1994)
*cis*-dioxolane	Agonist. Efficacy on AA and ↑cAMP pathways. (CHO-rM_1_ cells)	Biased cAMP/AA over IP_3_	Gurwitz et al. (1994)
Pilocarpine	Agonist. Efficacy on IP_3_, Ca^2+^, AA, ↑cAMP, G_i/o_, β-arrestin pathways (CHO-hM_1_ or rM_1_ cells)	Non-biased	Gurwitz et al. (1994), Thomas et al. (2008), and Davis et al. (2009)
Arecoline	Agonist. Efficacy on G_q_, IP_3_, AA, ↑cAMP, G_i/o_, β-arrestin pathways (CHO-hM_1_ or rM_1_ cells)	Non-biased	Gurwitz et al. (1994) and Thomas et al. (2008)
Cevimeline	Agonist. Efficacy on IP_3_, AA, ↑cAMP pathways (CHO-rM_1_ cells)	Non-biased	Gurwitz et al. (1994)
Xanomeline	Agonist. Efficacy on IP_3_ and β-arrestin pathways (CHO-hM_1_ cells)	Non-biased	Davis et al. (2009)
McN-A-343	Agonist. Efficacy on IP_3_, AA, ↑cAMP pathways (CHO-rM_1_ cells)	Non-biased	Gurwitz et al. (1994)
*cis*-AF30	Agonist. Efficacy on IP_3_, AA, ↑cAMP pathways (CHO-rM_1_ cells)	Non-biased	Gurwitz et al. (1994)
AC-42	Bitopic agonist. Efficacy on G_q_, IP_3_, ↑cAMP, β-arrestin pathways (CHO-hM_1_ cells)	Biased without efficacy for Gα_i/o_	Thomas et al. (2008) and Davis et al. (2009)
77-LH-28-1	Bitopic agonist. Efficacy on Gq, IP_3_, ↑cAMP pathways (CHO-hM1 cells)	Biased without efficacy for Gα_i/o_	Thomas et al. (2008)
AC-260584	Bitopic agonist. Efficacy on IP_3_, Ca^2+^ pathways (CHO-hM_1_ and HEK-hM_1_ cells)	Biased G protein over β-arrestin	Davis et al. (2009) and Davis et al. (2010a)
TBPB	Bitopic agonist. Efficacy on Ca^2+^, ERK1/2 pathways (CHO-hM_1_ and HEK-hM_1_ cells)	Biased G protein over β-arrestin	Davis et al. (2010b) and Keov et al. (2014)
VU0357017	Bitopic agonist. Efficacy on Ca^2+^, ERK1/2 pathways. (CHO-rM_1_ cells)	Biased G protein over β-arrestin	Digby et al. (2012)
VU0364572	Bitopic agonist. Efficacy on Ca^2+^, IP_3_, ERK1/2 pathways. (CHO-rM_1_ cells)	Biased. G protein over β-arrestin	Digby et al. (2012)
BQCA	PAM. Potentiates signaling on IP_3_, ERK1/2, G_s_, G_i/o_, G_q_, G_12_, ↑cAMP, β-arrestin pathways (CHO-hM_1_)	Non-biased	Canals et al. (2012) and Yeatman et al. (2014)
MIPS1645	PAM. Potentiates signaling on IP_3_, ERK1/2 and β-arrestin pathways (CHO-hM_1_)	Non-biased	van der Westhuizen et al. (2018)
MIPS1745	PAM. Potentiates signaling on IP_3_, ERK1/2 and β-arrestin pathways (CHO-hM_1_)	Non-biased	van der Westhuizen et al. (2018)
MIPS1780	PAM. Potentiates signaling on IP_3_, ERK1/2 and β-arrestin pathways (CHO-hM_1_)	Non-biased	van der Westhuizen et al. (2018)
VU6004256	PAM. Potentiates signaling on Ca^2+^, β-arrestin pathways. (CHO-hM_1_)	Biased Ca^2+^ and β-arrestin without internalisation	Rook et al. (2017)
VU0029767	PAM. Potentiates signaling on IP_3_ but not phosphatidylbutanol (CHO-rM1)	Biased PLC over PLD	Marlo et al. (2009)
VU0405645	PAM. Potentiates signaling on IP_3_ but not phosphatidylbutanol (CHO-rM1)	Biased PLC over PLD	Moran et al. (2019)
Pirenzepine	Antagonist. Efficacy on ERK1/2, β-arrestin, CREB pathways. (OC-033-rM1, HEK-CRISPR/Cas9-Δβ-arrestin, rat DRG cells)	Biased β-arrestins over G proteins	Sabbir and Fernyhough (2018)
Muscarinic toxin 7	Antagonist. Efficacy on ERK1/2, β-arrestin, CREB pathways. (OC-033-rM1, HEK-CRISPR/Cas9-Δβ-arrestin, rat DRG cells)	Biased β-arrestins over G proteins	Sabbir and Fernyhough (2018)

Abbreviations: AA, Arachidonic acid; cAMP, cyclic adenosine monophosphate; IP_3_, inositol trisphosphate; ERK, extracellular signal-regulated kinase.

At the M_4_ mAChR, to date, seven agonists have been systematically and quantitatively assayed for potential biased signaling across measures of cAMP inhibition and cAMP accumulation in CHO cells stably expressing the M_4_ mAChR and for IP_3_ accumulation in human embryonic kidney (HEK) cells co-expressing Gα_15_ and the M_4_ mAChR (Figueroa et al., 2009). There was no evidence of agonist-directed signaling for oxotremorine-M, carbachol, McN-A-343, S-aceclidine, R-aceclidine, arecoline or pilocarpine at any of the pathways tested (Table 2). A similar study assessed methacholine, oxotremorine-M, arecoline, bethanacol, oxotremorine or pilocarpine for bias in modulating cAMP inhibition vs. cAMP accumulation at the M_4_ mAChR, but found no quantitative differences in relative responses (Table 2) (Mistry et al., 2005). Due to the lack of apparent bias for mAChR agonists in these well-documented signaling assays, further studies looking for biased orthosteric agonists have not been pursued.

**TABLE 2:** Ligands screened, pharmacology assessed and biased profiles determined for a range of orthosteric, bitopic and allosteric ligands at the M_4_ mAChR. Different combinations of ligands and pathways have been used to assess a range of mAChR ligands for biased signaling. Biased signaling has been assessed in recombinant Chinese hamster ovary (CHO) or Human embryonic kidney (HEK) cells stably expressing the human (hM_4_) mAChRs.

Ligand	Pharmacology	Bias Profile	References
Carbachol	Agonist. Efficacy on ↑cAMP, ↓cAMP, IP_3_ pathways (CHO-hM_4_ and HEK-Gα_15_-hM_4_ cells)	Non-biased	Figueroa et al. (2009)
Oxoremorine-M	Agonist. Efficacy on ↑cAMP, ↓cAMP, IP_3_ pathways (CHO-hM_4_ and HEK-Gα_15_-hM_4_ cells)	Non-biased	Figueroa et al. (2009) and Mistry et al. (2005)
Methacholine	Agonist. Efficacy on ↑cAMP, ↓cAMP pathways (CHO-hM_4_)	Non-biased	Mistry et al. (2005)
Oxotremorine	Agonist. Efficacy on ↑cAMP, ↓cAMP pathways (CHO-hM_4_ cells)	Non-biased	Mistry et al. (2005)
Pilocarpine	Agonist. Efficacy on ↑cAMP, ↓cAMP pathways (CHO-hM_4_ cells)	Non-biased	Figueroa et al. (2009) and Mistry et al. (2005)
Arecoline	Agonist. Efficacy on ↑cAMP, ↓cAMP pathways (CHO-hM_4_ cells)	Non-biased	Mistry et al. (2005)
Bethanacol	Agonist. Efficacy on ↑cAMP, ↓cAMP pathways (CHO-hM_4_ cells)	Non-biased	Mistry et al. (2005)
S-aceclidine	Agonist. Efficacy on ↑cAMP, ↓cAMP, IP_3_ pathways (CHO-hM_4_ and HEK-Gα_15_-hM4 cells)	Non-biased	Figueroa et al. (2009)
R-aceclidine	Agonist. Efficacy on ↑cAMP, ↓cAMP, IP_3_ pathways (CHO-hM_4_ and HEK-Gα_15_-hM_4_ cells)	Non-biased	Figueroa et al. (2009)
McN-A-343	Agonist. Efficacy on ↑cAMP, ↓cAMP, IP_3_ pathways (CHO-hM_4_ and HEK-Gα_15_-hM_4_ cells)	Non-biased	Figueroa et al. (2009)
LY2033298	PAM. Potentiates GTPγS binding, ERK1/2, Ca^2+^, GSK-3β signaling pathways (CHO-hM_4_ cells)	Potential for bias. GTPγS, ERK1/2 and GSK-3β pathways	Chan et al. (2008) and Leach et al. (2010)

Abbreviations: AA, Arachidonic acid; cAMP, cyclic adenosine monophosphate; IP_3_, inositol trisphosphate; ERK, extracellular signal-regulated kinase.

#### Biased Allosteric Agonists

In CHO cells expressing the M_1_ mAChR, carbachol increases calcium levels, ERK1/2 phosphorylation, β-arrestin 2 translocation and causes receptor internalization (Davis et al., 2010b). In the same cell system, AC260584 and TBPB (Figure 4), which are M_1_ mAChR bitopic ligands that span both the orthosteric and allosteric sites, increased calcium levels and ERK1/2 phosphorylation, but did not translocate β-arrestin or cause receptor internalization (Table 1) (Davis et al., 2010b). Similarly, the bitopic ligands, VU0357017 and VU0364572 (Figure 4), also increased calcium and ERK1/2 phosphorylation in CHO cells overexpressing M_1_ mAChR, but they did not recruit β-arrestin (Table 1) (Digby et al., 2012). These results suggest that traditional orthosteric ligands, such as carbachol, activate all signaling pathways available to the receptor, whereas compounds that interact with the allosteric site, can direct their signal activation away from β-arrestin recruitment, receptor internalization and subsequent downstream signaling pathways. *In vivo* work with VU0357017 and VU0364572 showed that both compounds improved hippocampal dependent memory but failed to decrease amphetamine-induced hyper-locomotion in rats (Digby et al., 2012). A result also suggesting a link between G protein activation at the M_1_ mAChR and hyperlocomotion responses in rats, however, further work to confirm such a link is required.

#### Biased Antagonists

Pharmacologically distinct effects on different measures of cellular function are not restricted to mAChR agonists, with mAChR antagonists now linked to selective effects on intracellular signaling. Prolonged treatment (1–1.5 h) of M_1_ mAChR expressing cell lines with the mAChR antagonists muscarinic toxin 7 (MT7) or pirenzepine (Figure 4), increases ERK1/2 phosphorylation in acidic fractions (pH ∼3) and CREB phosphorylation in several different cell lines; an effect not replicated with the agonist carbachol (Table 1) (Sabbir and Fernyhough, 2018). In Δβ-arrestin 1/2 CRISPR/Cas-9 knockout HEK293 cells, CREB and ERK1/2 phosphorylation by pirenzepine and MT7 is lost, suggesting that this pathway requires β-arrestins (Sabbir and Fernyhough, 2018). Activation of this β-arrestin-ERK1/2-CREB signaling pathway increases neurite outgrowth in cultured primary dorsal root ganglion neurons (Sabbir and Fernyhough, 2018), suggesting that this signaling pathway has some physiological relevance. Further work to confirm this result and to explore whether other mAChR antagonists exhibit the same ERK1/2 phosphorylation effects may yield some interesting biased compounds that could be further developed for therapeutic benefit.

#### Biased Allosteric Modulators

With the recent advances in allosteric drug discovery, some mAChR allosteric modulators have been assessed for the degree to which they can induce bias by altering the pattern of signaling of orthosteric ligands. BQCA potentiates the responses to carbachol on cAMP production, ERK1/2 phosphorylation, IP_1_ accumulation, Gα_s_ activation, Gα_i__1/2_ activation, Gα_q_ activation, Gα_12_ activation and β-arrestin recruitment without altering the relative activation of the different pathways (Table 1) (Canals et al., 2012; Yeatman et al., 2014). Similarly, the structurally diverse M_1_ PAMs, MIPS1674, MIPS1745 and MIPS1780 induce equivalent potentiation of ACh-mediated IP_1_ accumulation, ERK1/2 phosphorylation, and β-arrestin recruitment (van der Westhuizen et al., 2018). Mechanistically, this is consistent with enhancement of affinity as the driver of observed cooperativity for these compounds (van der Westhuizen et al., 2018). When BQCA was assessed at the M_1_ DREADD, it weakly potentiated the effects of the DREADD ligand clozapine-N-oxide, in measures of calcium mobilisation, IP_1_, ERK1/2 and cAMP signaling (Abdul-Ridha et al., 2013). Of note, although BQCA was a PAM of the potency of clozapine-N-oxide on the cAMP pathway, it was a NAM of the efficacy of clozapine-N-oxide on the cAMP pathway. In contrast, when ACh was used as the agonist BQCA was a PAM for both potency and efficacy on the cAMP pathway (Abdul-Ridha et al., 2013). Thus, BQCA is a selective biased modulator of clozapine-N-oxide at the M_1_ DREADD. The mechanistic simplicity of the cooperativity between BQCA and ACh at M_1_ mAChRs, at all pathways investigated to date, makes this compound a good "reference" modulator for future studies.

In contrast to BQCA, biased modulation has been observed with the PAMs VU6004256, VU0029767 and VU0405645 (Figure 4) at the M_1_ mAChR (Table 1) (Marlo et al., 2009; Rook et al., 2017; Moran et al., 2019). In the case of VU6004256, comparison of this PAM with PF06764427 revealed that both PAMs potentiated ACh-mediated calcium fluxes and β-arrestin recruitment, however, PF-06764427 promoted M_1_ mAChR internalisation whereas VU6004256 did not (Rook et al., 2017). Further studies revealed that the PAMs VU0090157 and VU0453595 activate both phospholipase C (PLC) and phospholipase D (PLD) pathways, whereas VU0029767 and VU0405645 coupled only to PLC pathways but not PLD pathways (Marlo et al., 2009; Moran et al., 2019). Furthermore, VU0453595 potentiated carbachol-mediated field excitatory postsynaptic potentials (fEPSPs) in mouse prefrontal cortex containing coronal slices, but VU0405645 did not, suggesting that compounds that select for PLC pathways over PLD pathways can actually be detrimental to the formation of long term depression in the prefrontal cortex (Moran et al., 2019).

At the M_4_ mAChR, LY2033298 has a robust allosteric agonist profile toward G protein activation, ERK1/2 phosphorylation and the GSK-3β signaling pathway. Whilst LY2033298 displayed various degree of agonist activity in most signaling assays, no allosteric agonism for the calcium signaling pathway was detected (Table 2) (Chan et al., 2008; Leach et al., 2010). In receptor internalization assays, LY2033298 had a small degree of allosteric agonist activity but a large degree of allosteric potentiation, more so than for the any other signaling assays investigated. Whilst the potential for bias was not directly quantified in these studies, the results hinted at the potential for biased modulation at the M_4_ mAChR. With the development of many new M_4_ mAChR PAMs, further work to explore the potential for biased modulation of the M_4_ mAChR may yield new and exciting results in this field.

## Important Signaling Pathways to Target With Biased Allosteric Modulators

**FIGURE 5:**
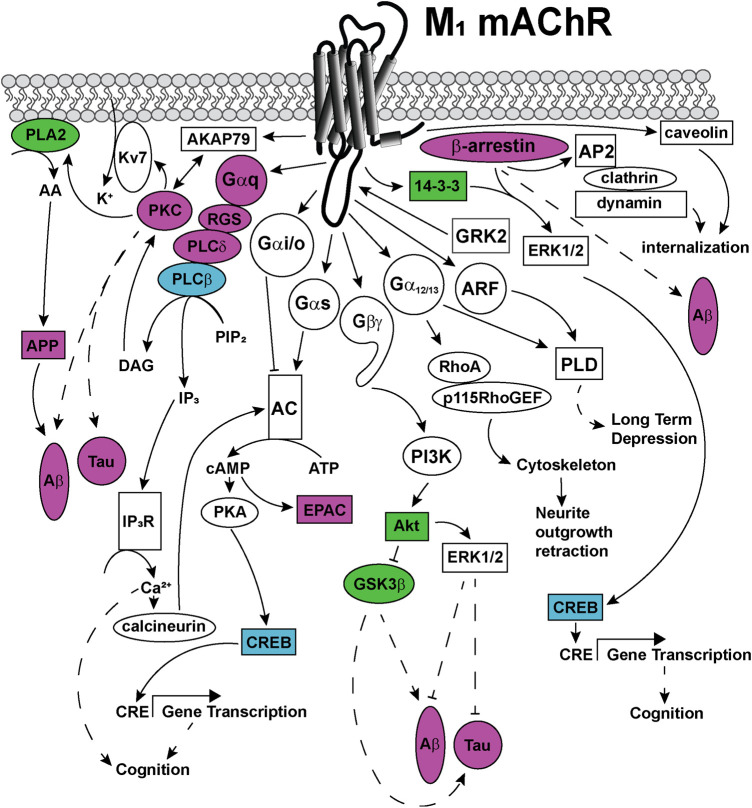
Signaling pathways coupled to the M_1_ mAChR. Upon activation, the M_1_ mAChR principally couples to Gα_q_ proteins to activate phospholipase C (PLC) enzymes to catalyze the membrane lipid substrate, phosphatidyl inositol 4,5-bisphosphate (PIP_2_) into inositol trisphophate (IP_3_) and diacylglycerol (DAG). DAG activates protein kinase C (PKC), which is anchored to the M_1_ mAChR via a direct interaction with the scaffold protein, A-kinase anchoring protein (AKAP)-79. This AKAP-PKC complex activates the potassium M-channel (Kv7/KCNQ), permitting the influx of potassium (K^+^) into neurons to regulate neuronal excitability. IP_3_ activates the IP_3_ receptor (IP_3_R) on the sarcoplasmic reticulum, releasing calcium (Ca^2+^) from the intracellular stores to the cytosol. Ca^2+^ activates intracellular signaling proteins, such as calcineurin, which can in turn activate some adenylate cyclase (AC) isoforms. PKC activates phospholipase A (PLA)_2_, which increases cytosolic arachidonic acid (AA), releasing amyloid precursor proteins (APP) and driving the formation of toxic amyloid-β (Aβ) plaques in Alzheimer's disease. The M_1_ mAChR also couples to Gα_s_ and Gα_i/o_ proteins, which can directly activate or inhibit AC, respectively. Activated AC converts cytosolic adenosine triphosphate (ATP) to the second messenger, cyclic adenosine monophosphate (cAMP). cAMP can in turn, activate signaling proteins such as protein kinase A (PKA) or exchange protein directly activated by cAMP (EPAC). PKA phosphorylates and activates cAMP response element binding protein (CREB), which increases gene transcription from cAMP response elements (CRE). Genes with CRE promoter regions are linked to improved cognition. The M_1_ mAChR couples to Gα_12/13_ proteins, which activate Ras homolog family member A (RhoA), Rho guanine nucleotide exchange factor 1 (p115RhoGEF), resulting in cytoskeletal rearrangements and neurite outgrowth retraction. Phospholipase D (PLD) is activated by either Gα_12/13_ proteins or the small GTPase adenosine diphosphate-ribosylation factor (ARF) proteins, to induce long-term depression. The Gβγ-subunits are also linked to signal transduction, through activation of phosphoinosotide 3-kinase (PI3K) and protein kinase B (Akt). Akt activates extracellular signal-regulated kinase (ERK) 1/2 which is linked to decreases in Aβ levels and decreases in Tau protein phosphorylation. Akt inhibits the activity of glycogen synthase kinase (GSK) 3β, a kinase that is associated with increases in Aβ and Tau hyperphosphorylation in Alzheimer's disease. M_1_ mAChR signaling is regulated by phosphorylation of the receptor at serine and threonine residues located in intracellular loops and carboxy-terminal tail, by kinases including GRK2. GRK2 phophorylation promotes the recruitment of β-arrestin scaffold proteins that promote M_1_ mAChR internalisation via clathrin-coated pits through interactions with the adaptor complex, AP2 protein, clathrin and dynamin. Additional M_1_ mAChR internalization may occur via recruitment of caveolins, through clathrin-independent pathways. Recruitment of scaffold proteins such as, β-arrestin and 14-3-3 are also linked to ERK1/2 activation and increases in transcription from CRE elements. Dashed arrows indicate pathways requiring further work to characterise the signaling proteins involved in the pathway. Solid arrows represent experimentally determined interactions. Proteins that are disrupted in Alzheimer's disease are colored in purple, those disrupted in schizophrenia are colored in blue and those disrupted in both diseases are colored in green.

**FIGURE 6:**
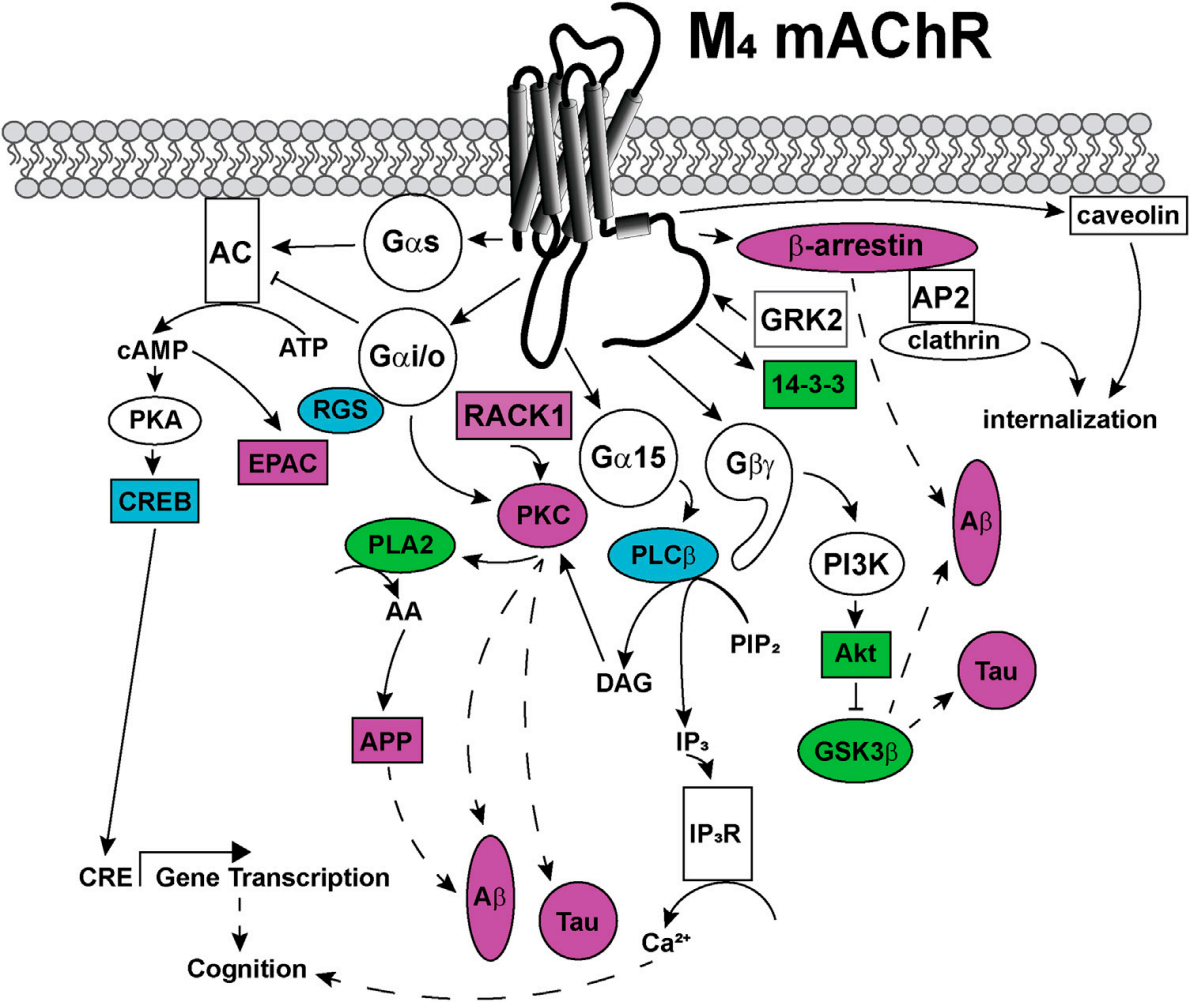
Signaling pathways coupled to the M_4_ mAChR. Upon activation, the M_4_ mAChR is principally coupled to Gα_i/o_ proteins that inhibit adenylate cyclase (AC) to decrease cytosolic cyclic adenosine monophosphate (cAMP) levels. The M_4_ mAChR also couples to Gα_s_ proteins, when activated by high (100 μM) concentrations of agonists. Gα_s_ directly activates adenylate cyclase to convert cytosolic adenosine triphosphate (ATP) to cAMP, a second messenger that can activate protein kinase A (PKA) and exchange protein directly activated by cAMP (EPAC). PKA phosphorylates and activates cAMP response element binding protein (CREB), which increases gene transcription from cAMP response elements (CRE). Phospholipase A (PLA)_2_ is activated by the M_4_ mAChR via a mechanism involving Gα_i/o_ and protein kinase C (PKC). PLA_2_ increases intracellular arachidonic acid (AA) levels, which promotes amyloid precursor protein (APP) release, amyloid-β (Aβ) plaque formation and Tau protein hyperphosphorylation in Alzheimer's disease. Phosphoinositol 3-kinase (PI3K) is activated by Gβγ-subunits, and PI3K activates protein kinase B (Akt), which in turn inhibits glycogen synthase kinase (GSK) 3β affecting the production of toxic Aβ plaques and Tau hyperphosphorylation. Receptor for activated C-kinase (RACK) one is recruited to the M_4_ mAChR and scaffolds PKC, which may be involved in Aβ plaque formation and Tau hyperphosphorylation. Gα_15_ coupling links M_4_ mAChR activation with increases in intracellular IP_3_ levels and related signaling responses. The M_4_ mAChR is phosphorylated by GRK2 and recruits β-arrestin to terminate G protein-mediated signaling events. M_4_ mAChR cell surface expression levels are regulated by internalization, either by recruitment of β-arrestins and internalization via clathrin-coated pits or by caveolin-dependent mechanisms. Dashed arrows indicate pathways requiring further work to characterise the signaling proteins involved in the pathway. Solid arrows represent experimentally determined interactions. Proteins that are disrupted in Alzheimer's disease are colored in purple, those disrupted in schizophrenia are colored in blue and those disrupted in both diseases are colored in green.

Like most GPCRs, the M_1_ and M_4_ mAChRs couple to a wide array of signal transduction pathways, as can be seen in Figures 5, 6. These include those linked to phospholipase C (PLC), calcium release, phospholipase D (PLD), phospholipase A_2_(PLA_2_), cAMP increases and decreases, and mitogen-activated protein kinase pathways (Felder, 1995; Nathanson, 2000). Some of these pathways have well-described roles in learning, memory and synaptic plasticity, and are implicated in the symptomology of neurological diseases, while others require further characterisation to identify potential avenues for the development of new biased ligands for the mAChRs.

### Gα_q_-Inositol Trisphosphate (IP_3_)-Calcium-Protein Kinase C Signaling

The M_1_ mAChR is principally coupled to Gα_q/11_ linking the receptor to phospholipase C (PLC), inositol trisphosphate (IP_3_), diacylglycerol (DAG), protein kinase C (PKC) and calcium signaling pathways (Figure 5). The Gα_q_-PLC signaling pathway is also linked to the activation of cAMP, via a mechanism involving IP_3_-calcium release and calmodulin (Felder et al., 1989). [^35^S]GTPγS assays in hippocampal and cortical cultures revealed that direct activation of the Gα_q/11_ proteins was abolished in the M_1_ mAChR knockout mouse (Porter et al., 2002; Wess, 2004), confirming a role for Gα_q_ for the M_1_ mAChR. In M_1_ mAChR knockout mouse primary cortical neurons, inositol phosphate (PI) hydrolysis was also dramatically reduced (Hamilton and Nathanson, 2001; Wess, 2004), linking Gα_q_ and PI signaling pathways to the M_1_ mAChR in areas of the brain that are important in cognition. Activation of this signaling pathway may be particularly relevant for improving cognition, because influxes of calcium into neurons increase the activity of calcium-dependent adenylate cyclases, which are linked to generating short term memory, coding for memories that last minutes to days to weeks (Kandel, 2012).

Targeting Gα_q/11_ signaling pathways may also be relevant for treating the symptoms of schizophrenia. Gene expression microarray data comparing genes from schizophrenic and non-schizophrenic patients identified decreases in PLCβ1 in the dorsolateral prefrontal cortex of schizophrenic patients (McOmish et al., 2008a). In addition, PLCβ1 knockout mice display abnormal cortical development, are hyperactive, have reduced pre-pulse inhibition responses and have diminished spatial memory in the Morris water maze test (McOmish et al., 2008a). Therefore, in schizophrenia the decreases in PLCβ1 may influence the downstream signaling pathways by way of decreased IP_3_ production, decreased calcium release and decreases in cognition.

The Gα_q/11_-mediated signaling pathway may also be relevant to treating Alzheimer's disease. In the prefrontal cortex of Alzheimer’s disease brains, there is decreased activity of PKC and glycogen synthase kinase (GSK) 3β (Tsang et al., 2007; Medeiros et al., 2011). The reduction in PKC and GSK3β activity induces Tau protein hyper-phosphorylation and amyloid-β processing (Medeiros et al., 2011). Receptors for activated C kinase 1 (RACK1) is reported to directly interact with the M_1_ mAChR (Borroto-Escuela et al., 2011a), and the expression of RACK1 is also decreased in the cortex of Alzheimer's disease post-mortem brain tissues compared to healthy aged brain tissues (Battaini et al., 1999). This loss in RACK1 may contribute to the decrease in PKC activity by disrupting the subcellular localisation of PKC. Overexpression of amyloid-β in cortical neuron cultures disrupts the membrane distribution of RACK1 (Liu et al., 2011), thus it is likely that disruption of the RACK1-PKC complex by amyloid-β contributes to the pathology of Alzheimer's disease. The neurofibrillary tangles within the neurons of Alzheimer's disease brains also accumulate phospholipase Cδ (Shimohama et al., 1991), which likely hinders the functioning of this PLC. Furthermore, the duration of signaling from the G protein is regulated by direct interactions between the Gα subunits and regulator of G proteins signaling (RGS) proteins. The M_1_ mAChR directly interacts with RGS2 and RGS8 (Bernstein et al., 2004; Itoh et al., 2006; Borroto-Escuela et al., 2011a), where both RGS2 and RGS8 can interact with Gα_q_ and the third intracellular loop of the M_1_ mAChR to switch-off Gα_q_ signaling (Bernstein et al., 2004; Itoh et al., 2006). Interestingly, as observed with other proteins involved in the Gα_q_ signaling pathway, RGS2 levels are decreased in Alzheimer’s patients (Hadar et al., 2016). Together, these findings suggest that the pathological increases in amyloid-β and neurofibrillary tangles that are seen in Alzheimer's disease reduce Gα_q_-mediated signaling events, which may have implications for the treatment of the disease.

Although the expression level of the M_1_ mAChR is unchanged in Alzheimer’s disease brains when compared to age matched controls, the functionality of the M_1_ mAChR is possibly compromised (Jope et al., 1994; Bradley et al., 2017). Several studies have explored the binding and signaling properties of M_1_ mAChRs in post mortem tissue samples from Alzheimer's disease and healthy aged brains. In radioligand binding studies, high and low affinity binding components are often observed with increasing concentrations of competing agonists, which are associated with the G protein-coupled and uncoupled states of the receptor, respectively. In Alzheimer's disease post-mortem tissues there is a loss in the high affinity binding component for carbachol when either [^3^H]-N-methylscopolamine or [^3^H]-pirenzepine are used as the radioligand, suggesting disruption in coupling to Gα proteins (Ferrari-DiLeo and Flynn, 1993). Furthermore, carbachol or oxotremorine M-stimulated PIP_2_ hydrolysis is decreased in cortical membranes of Alzheimer's disease patients, and can be blocked with the M_1_ mAChR antagonist, pirenzepine (Ferrari-DiLeo and Flynn, 1993; Jope et al., 1994). The functionality of PLC activity from Alzheimer's disease brains was unchanged compared to that in unaffected brain; therefore, it is likely due to inefficient activation of the PLC by Gα_q_ rather than due to a deficiency in PLC itself (Ferrari-DiLeo and Flynn, 1993; Jope et al., 1994). More recently, direct assessment of G protein activation by [^35^S]-GTPγS binding assays, demonstrated that G protein activation was the same in Alzheimer's disease tissues compared to aged matched controls (Bradley et al., 2017). Thus G protein activation is equivalent in healthy and diseased brain tissues. Further work to explore the coupling of the M_1_ mAChR with G proteins and the efficiency of Gα activation is required to determine the nature of the disruption of M_1_ mAChR signal transduction in Alzheimer’s disease.

### Gα_q_-A Kinase Anchoring Protein (AKAP)79-PKC-Potassium Channels

Potassium M-currents occur through the Kv7 (KCNQ) potassium channels, and regulate a slow ingress of potassium ions into neurons, to regulate the excitability of neurons (Passmore et al., 2012). In M_1_ mAChR knockout mice, inhibition of the potassium M-current was observed in sympathetic neurons (Hamilton et al., 1997). Further work examining the potassium M-current in dentate gyrus granule cells and hippocampal CA1 pyramidal cells indicate that the M_1_ mAChR may enhance rather than supress M-currents in different neuronal populations (Carver and Shapiro, 2019). This enhancement of the M-current is proposed to occur via phosphatidylinositol 4,5-bisphosphate (PIP_2_) synthesis from phosphatidylinositol 4-phosphate or phosphatidylinositol 5-phosphate in the dentate gyrus granule cells (Carver and Shapiro, 2019). In CA1 pyramidal neurons, the M-current was suppressed via a mechanism involving the depletion of PIP_2_ (Carver and Shapiro, 2019). The M_1_ mAChR directly interacts with AKAP79 (Borroto-Escuela et al., 2011a), which anchors PKC to the receptor. The M_1_ mAChR-AKAP79-PKC complex phosphorylates and activates potassium channels to regulate the M-current in post-synaptic cells (Hoshi et al., 2010). This mechanism is important for regulating neuronal excitability. Together, these results suggest that the M_1_ mAChR is involved in cell-type specific regulation of neuronal excitability state through the regulation of potassium channels.

### Gα_q_-Phosphoinostiol 3-Kinase (PI3K)-Src-ERK1/2

Extracellular regulated kinases (ERK) are members of the mitogen activated protein kinase (MAPK) family. ERK1/2 are the most abundant isoforms of ERK expressed in the brain (Mazzucchelli et al., 2002). ERK1 and ERK2 knockout mouse studies directly link ERK1/2 activation with enhanced cognition (Mazzucchelli et al., 2002; Satoh et al., 2007). The mechanism underlying this improvement in memory may occur via ERK1/2 phosphorylation and subsequent activation of CREB, which is linked to increases in memory formation (Mazzucchelli et al., 2002; Kandel, 2012; Xia and Storm, 2012).

When ACh levels are increased in rats with the acetylcholinesterase inhibitor, physostigmine, enhanced levels of ERK1/2 phosphorylation are observed in the hippocampus and the cortex (Rosenblum et al., 2000). Physostigmine-induced ERK1/2 phosphorylation in the cortex and hippocampus are blocked with the administration of atropine, suggesting that this response occurs via activation of mAChRs (Rosenblum et al., 2000; Xia and Storm, 2012). In whole anesthetized rats, long term potentiation (LTP) is induced by tetanic stimulation in the dentate gyrus (Rosenblum et al., 2000). This LTP is blocked in animals that are pre-treated with the mitogen activated protein kinase kinase (MEK) inhibitor, PD98059 (Rosenblum et al., 2000). Together these results suggest that activation of the ERK1/2 signaling pathway in the brain is involved in the formation of memory and that ERK1/2 activation can occur via the mAChRs in the rat brain. In the M_1_ mAChR knockout mouse, ERK1/2 signaling was not evident in primary cortical neurons or CA1 hippocampal neurons from newborn pups (Berkeley et al., 2001). This suggests that activation of ERK1/2 downstream of the M_1_ mAChR is important, and may represent an ideal pathway to activate to improve cognition.

ERK1/2 activation by the M_1_ mAChR likely involves both G protein-dependent and β-arrestin-dependent pathways. In primary cortical cultures or African green monkey kidney (COS7) cells over-expressing the M_1_ mAChR, carbachol-stimulated ERK1/2 phosphorylation is blocked by PI3K (LY294002) and Src (PP1) inhibitors, indicating a role for both of these kinases in the M_1_ mAChR-mediated ERK1/2 activation pathway (Rosenblum et al., 2000). When the same cells were treated with the calcium chelators, BAPTA-AM, EGTA or a PKC inhibitor (BIM1), ERK1/2 phosphorylation was still observed (Rosenblum et al., 2000). Other studies suggest that the M_1_ mAChR activates ERK1/2 by Gα_q_-PKC dependent manner in CHO or COS7 cells (Hawes et al., 1995), or via Gα_o_ in a pertussis toxin-sensitive manner in CHO cells (van Biesen et al., 1996). M_1_ mAChR activation of ERK1/2 via a PI3K-dependent pathway may be important because activation of a PI3K-Akt-ERK1/2-dependent pathway is important for protecting against Alzheimer's disease (Rai et al., 2019). Given that these pathways may contribute to the symptoms of neurological diseases, selective targeting of ERK1/2 pathways may also be an important pathway to target with biased modulators.

### Gα_i/o_-Gβγ-PI3K-ERK1/2

Less is known about the mechanism of ERK1/2 activation by the M_4_ mAChR. However, parallels may be drawn from the M_2_ mAChR, which is also a Gα_i/o_ coupled receptor. In COS7 cells over-expressing the M_2_ mAChR, carbachol increases ERK1/2 phosphorylation, which is blocked with a PI3K inhibitor (wortmannin) and the Gβγ subunit inhibitor (βARK-ct) (Lopez-Ilasaca et al., 1997). In M_4_ mAChR knockout mice, ERK1/2 signaling was unaffected; suggesting that activation of ERK1/2 is not a critical pathway downstream of the M_4_ mAChR (Berkeley et al., 2001; Wess, 2004). Allosteric modulation of the M_4_ mAChR with VU0152100 inhibits D_1_ dopamine receptor-induced ERK1/2 activation in rat striatum and medial prefrontal cortex (Xue et al., 2015). Thus, the M_4_ mAChR exerts an inhibitory effect toward the ERK1/2 signaling pathway via crosstalk with the D_1_ dopamine receptor. Western blots of post-mortem brain tissue of patients with schizophrenia and mood disorders determined that in the prefrontal cortex, there are decreased protein expression levels of B-raf, MEK1, MEK2, RSK1, CREB, and Rap1, all members of the MAPK signaling pathway (Yuan et al., 2010; Funk et al., 2012). This indicates that in schizophrenia and mood disorders, an under-stimulation of ERK1/2 signaling pathway may be involved in the symptomology of the disease, further supporting the notion that activation of the ERK1/2 pathway may be beneficial for treating neurological disorders.

### Gα_12/13_-RhoA-Phospholipase D (PLD)

Interestingly, the M_1_ mAChR is reported to directly interact with Gα_12/13_ proteins (Borroto-Escuela et al., 2011a). Gα_12/13_ proteins activate Rho proteins, which are involved in the rearrangement of the actin cytoskeleton and regulate membrane trafficking (Ridley et al., 1992; Ridley and Hall, 1992; Ridley 2001; Suzuki et al., 2009). Activation of Gα_13_ is linked to the retraction of neurite outgrowths in adult sensory neurons, an effect that is reversed with the M_1_ mAChR antagonists, pirenzepine and muscarinic toxin 7 (Sabbir and Fernyhough, 2018). Gα_12/13_ proteins are also linked to the activation of PLD (Plonk et al., 1998). Although a direct link between the M_1_ mAChR activation of Gα_12/13_ and subsequent PLD activation is lacking, such a link has been established for the related M_3_ mAChR, which activates PLD via Gα_12/13_ proteins in HEK293 cells (Rumenapp et al., 2001). Given the link between Gα_12/13_, PLD activation and the important role of PLD in long-term depression in the prefrontal cortex, further work to determine whether or not the M_1_ mAChR biased allosteric modulators can selectively activate Gα_12/13_ signaling pathways may provide vital information regarding the direction for the development of future M_1_ mAChR targeted therapeutics.

### Adenosine Diphosphate Ribosylation Factor (ARF)-PLD

Mass spectrometry experiments suggest that the M_1_ mAChR interacts with ADP ribosylation factors (ARF) 1, 3, 5 and 6, which are small Ras-family, GTP binding proteins (Borroto-Escuela et al., 2011a). Although these interacting partners have been identified for the M_1_ mAChR, there are no subsequent studies to date confirming these interactions with the M_1_ mAChR. Studies looking at the related M_3_ mAChR demonstrate that ARF 1 and 6 directly interact with the M_3_ mAChR to activate PLD (Mitchell et al., 2003). This interaction requires the asparagine residue of the NPxxY motif at the end of transmembrane 7 (Borroto-Escuela et al., 2011b). Given the importance of the PLD pathway in long-term depression in the prefrontal cortex and the potential for biased allosteric modulation of this pathway, ARFs may be an important family of proteins to monitor for novel mAChR ligand characterisation. Although the M_1_ and M_3_ mAChR are both Gα_q_-coupled related receptors, further work to confirm direct interactions between the M_1_ mAChR and ARFs is required. In addition, links between ARFs and PLD signaling at the M_1_ mAChR may provide a new avenue to exploit for biased allosteric modulators.

### Gα_i/o_-AC

Because the M_4_ mAChR is a Gα_i/o_-coupled receptor, one of its best characterised second messenger responses is the inhibition of adenylate cyclase, thus decreasing intracellular cAMP levels. There are five subtypes of Gα_i/o_ proteins, and the M_4_ mAChR specifically couples to the Gα_i2_ and Gα_oA_ and Gα_oB_ alpha subunits (Migeon et al., 1994; Migeon et al., 1995; Borroto-Escuela et al., 2011a). Mass spectrometry data also identified a possible interaction between the M_4_ mAChR and Gα_i3_ (Borroto-Escuela et al., 2011a), however, the functional consequences of this interaction remain to be confirmed. The M_4_ mAChR also interacts with RGS4, which is reported to be selective for regulating Gα_i_ activity (Roy et al., 2003; Borroto-Escuela et al., 2011a). Interestingly, DNA microarray analysis demonstrated a significant reduction in RGS4 expression levels in the prefrontal cortex of schizophrenic patients (Mirnics et al., 2001) and RGS4 expression is dysregulated in the PLCβ1 knockout mouse (McOmish et al., 2008b). This may provide a hint that the Gα_i/o_ signaling pathway is dysregulated in schizophrenia; however a direct link between the M_4_ mAChR-RGS4 and schizophrenia remains to be established. Interestingly, although activation of the M_4_ mAChR is normally linked to decreases in forskolin-stimulated cAMP levels, activation with high agonist concentrations can increase intracellular cAMP levels in CHO cells (Migeon and Nathanson, 1994; Migeon et al., 1995). Chronic activation of the M_4_ mAChR with the agonist McN-A-343 (100 μM; 18 h) also increases cAMP levels in CHO cells (Nevo et al., 1998). This possibly occurs through a “super-activation” mechanism involving Gβγ subunit signaling of the Gα_i/o_ proteins, which has been observed for mAChRs (Sunahara et al., 1996). This super-activation mechanism may be therapeutically relevant, as the lifetime of synthetic agonist ligands may impact the direction of cAMP levels in neurons. This is an important consideration for the design of synthetic M_4_ mAChR ligands to avoid overstimulation of receptors.

Some studies have also reported that the M_1_ mAChR can decrease cAMP signaling by coupling to Gα_i/o_ proteins. Direct activation of Gα_i1/2_, determined using [^35^S]-GTPγS binding assays, is observed for several orthosteric and bitopic mAChR ligands in CHO cells overexpressing M_1_ mAChR (Thomas et al., 2008). Of note, interactions between the M_1_ mAChR and the Gα_i/o_ proteins were not observed by mass spectrometry (Borroto-Escuela et al., 2011a). However, this may be explained by the faster dissociation rates of the Gα_i/o_ proteins from the M_1_ mAChR when compared with the dissociation rates of the Gα_q_ proteins (Ilyaskina et al., 2018). Further work is required to determine the potential physiological relevance of M_1_ mAChR coupling to Gα_i/o_ proteins and to inhibition of cAMP signaling.

### PI3K-Protein Kinase B (PKB/Akt)-GSK3β

The PI3K-Akt-GSK3β pathway is downregulated in schizophrenia (Karam et al., 2010; Emamian, 2012; Enriquez-Barreto and Morales, 2016; McGuire et al., 2017). Decreased levels of Akt and decreased inhibition of GSK3β are observed in the cortex and peripheral lymphocytes of human schizophrenic patients relative to controls (Emamian et al., 2004). The reduction of Akt expression levels may be linked to certain single nucleotide polymorphisms identified in a subset of schizophrenic patients, which cause a reduction in the transcription or translation of the *AKT1* gene (Emamian et al., 2004). Increased activity of Akt is observed in Alzheimer's disease brain tissues, resulting in an increase in the phosphorylation of the downstream proteins GSK3β(Ser9) (inhibits activity), Tau(Ser214) and mTor(Ser2448) (Pei et al., 2003; Griffin et al., 2005). Carbachol stimulation of an endogenously expressed mAChR in PC12 cells is linked to Akt phosphorylation (Wu and Wong, 2006). The carbachol-stimulated Akt phosphorylation is inhibited by pertussis toxin, suggesting that it occurs downstream of a Gα_i/o_-coupled mAChR (Wu and Wong, 2006). Few studies have investigated the GSK3β phosphorylation by mAChR ligands, however, this would be a pathway to try to target for the treatment of both Alzheimer's disease and schizophrenia.

### Gα_s_-AC-PKA-CREB

cAMP is a second messenger that activates PKA, exchange protein activated by cAMP (EPAC) and cAMP response element binding protein (CREB). When CREB is activated by PKA, mitogen-activated protein kinase (MAPK) or calcium/calmodulin-stimulated protein kinase (CaMK), CREB binds to cAMP response element promoter regions of DNA to increase gene transcription. This in turn, increases protein expression, increases synaptic strength and promotes long term memory storage (Davis et al., 1996; Montminy, 1997; Pittenger et al., 2006; Kandel, 2012). Long term memory can be blocked by overexpression of a phosphodeficient CREB(S133A) mutant in rat dorsolateral striatum (Pittenger et al., 2006; Brightwell et al., 2008). Thus, biased ligands that selectively enhanced the cAMP-CREB pathway could have potential to improve long term memory in patients with neurological disorders.

The M_1_ and M_4_ mAChRs are also reported to increase cAMP levels by coupling to Gα_s_ proteins in CHO cells (Burford and Nahorski, 1996; Canals et al., 2012). cAMP accumulation is observed in cells pre-treated with pertussis toxin (Gα_i/o_ inhibitor) and is blocked in cells treated with a Gα_s_ antiserum (Migeon et al., 1995; Burford and Nahorski, 1996). In addition, when the AC activator, forskolin, is co-administered with mAChR agonists, an increase in cAMP level is observed (Burford and Nahorski, 1996). Increases in cAMP in M_1_ mAChR expressing CHO cells are reported with both orthosteric and allosteric agonists (Thomas et al., 2008). There is, however, a lack of evidence showing a direct interaction between the M_1_ mAChR and Gα_s_. Data from mass spectrometry suggests that Gα_s_ interacts with the M_4_ mAChR but not the M_1_ mAChR (Borroto-Escuela et al., 2011a). Furthermore, at the M_1_ mAChR, [^35^S]-GTPγS assays using a Gα_s_ antibody to pull down activated Gα_s_ proteins, did not detect Gα_s_ activation (Thomas et al., 2008), and Forster resonance energy transfer (FRET) studies did not detect an interaction between the M_1_ mAChR and Gα_s_ (Ilyaskina et al., 2018). Together, these results may suggest that the M_1_ mAChR couples extremely weakly to Gα_s_, and may be a product of the overexpression of the M_1_ mAChR in a recombinant system. In CHO cells, recombinantly expressing the M_4_ mAChR, increases in cAMP levels are routinely reported; an effect that is reversed by overexpressing Gα_i2_ or Gα_o_ proteins (Migeon et al., 1995). Additionally, the M_4_ mAChR may activate cAMP through the Gβγ subunits depending on which AC isoforms are present in the cell (Varga et al., 1998). Interestingly, high agonist concentrations are required to increase cAMP levels at both the M_1_ and M_4_ mAChRs (Migeon et al., 1995; Burford and Nahorski, 1996), which makes the physiological relevance of cAMP signaling by the mAChRs questionable. Thus, further work to identify the mechanism and physiological relevance of Gα_s_ activation by the M_1_ and M_4_ mAChRs is required.

Changes in levels of cAMP pathway-associated proteins may be important in producing some symptoms of schizophrenia. In post-mortem schizophrenic brain tissues, changes in the expression level of proteins associated with the cAMP signaling pathway are indeed observed. Rap2, CREB and phosphodiesterase (PDE) 4B are either upregulated or downregulated, depending on the region of the brain that is investigated (Millar et al., 2005; Funk et al., 2012). Since schizophrenia is primarily considered a disorder of dopamine signaling, much of the evidence linking the cAMP pathway with schizophrenia is described within the context of D_1_ or D_2_ dopamine receptor activation (Wang et al., 2018). There is currently no direct evidence linking the mAChRs with cAMP activation to improve the symptoms of schizophrenia.

### β-Arrestin-ERK1/2

β-arrestin is a scaffold protein that can bind to members of the MAPK pathway, including cRaf, MEK and ERK1/2 (Song et al., 2009). Through scaffolding the relevant proteins together, β-arrestin activates the ERK1/2 signaling pathway. Following activation of Gα_q_ protein, the M_1_ mAChR is regulated by phosphorylation at serine and threonine residues in the intracellular loop three and carboxy-terminal tail by intracellular kinases. Phosphorylation of the M_1_ mAChR at Ser228/Ser273 by G protein-coupled receptor kinase (GRK) 2 results in translocation of β-arrestin 2 to the receptor, which in turn scaffolds the MAPK and results in ERK1/2 activation (Jung et al., 2017). Similar studies interrogating β-arrestin-ERK1/2 activation have not yet been performed for the M_4_ mAChR, but would be important for understanding M_4_ mAChR signaling. Since M_1_ mAChR-Gα_q_ pathways may be compromised in Alzheimer's disease, ERK1/2 activation to improve cognition could be achieved with β-arrestin-biased modulators. This could also be beneficial in reducing salivation and gastrointestinal disturbances that appear to occur downstream of Gα_q_ proteins in the M_1_ mAChR phosphodeficient mice (Bradley et al., 2020).

### G Protein-Coupled Receptor Kinase 2 (GRK2)-β-Arrestin-Internalisation

Most GPCRs are regulated by phosphorylation on their intracellular loops and carboxy-terminal tails by cellular kinases. These phosphorylation events uncouple GPCRs from G protein signaling and trigger receptor internalisation and downstream trafficking events. Intracellular scaffold proteins are subsequently recruited to phosphorylated GPCRs to assemble cellular proteins into specific signaling complexes. GRKs that phosphorylate GPCRs are one of the most studied GPCR kinases. The M_1_ and M_4_ mAChRs are phosphorylated in the third intracellular loop by GRK2 (Haga et al., 1996; Borroto-Escuela et al., 2011a; Jung et al., 2017). In the case of the M_1_ mAChR, GRK2 directly interacts with Gα_q_ to inhibit phosphoinositide signaling events (Willets et al., 2005), whilst for the M_4_ mAChR, GRK2 overexpression increases the rate of internalisation of the receptor (Holroyd et al., 1999; Vogler et al., 1999; Reiner and Nathanson, 2008; Borroto-Escuela et al., 2011a). Phosphorylation of GPCRs by GRKs increases β-arrestin translocation from the cytosol to the plasma membrane. The M_1_ mAChR rapidly recruits β-arrestin with a preference for β-arrestin 2 over β-arrestin 1 in RBL-2H3 or CHO cells expressing systems (Santini et al., 2000; Canals et al., 2012; Yeatman et al., 2014). Interestingly, both orthosteric and allosteric agonists promote β-arrestin recruitment to and internalisation of the M_1_ mAChR.

β-arrestin 2 is important in memory as evidenced by impaired fear conditioning responses in β-arrestin 2 knockout mice (Li et al., 2009). This suggests that β-arrestin 2 is important for amygdala based fear associative memory (Li et al., 2009). β-arrestin 1 and 2 are also implicated in Alzheimer’s disease, because the levels of both β-arrestin 1 and 2 are elevated in autopsied brains of Alzheimer's disease patients, when compared to age matched controls (Thathiah et al., 2013; Jiang et al., 2013). Overexpression of β-arrestin 2 in HEK 293-APP_695_ cells increased the secretion of amyloid-β, which was blocked when the cells were pre-treated with a γ-secretase inhibitor, L-685458 (Thathiah et al., 2013). The secretion of amyloid-β is also reduced in cells where the β-arrestin 2 gene is silenced, and in the β-arrestin 2 knockout mouse (Thathiah et al., 2013). Interestingly, there was no effect on the APP, with no change in expression levels with overexpression or silencing of β-arrestin 2 (Thathiah et al., 2013). β-arrestin 2 signaling may also be an important pathway to target for novel anti-schizophrenic therapies. In mouse models of schizophrenia (phencyclidine treated or NMDA receptor NR1 subunit knockdown mice), selective activation of β-arrestin 2 pathways with D_2_ dopamine receptor biased agonists reduced hyperlocomotion, restored pre-pulse inhibition, improved novel object recognition, improved social behaviors and reduced seizures (Park et al., 2016). Therefore, selectively activating β-arrestin 2-dependent signaling pathways may be beneficial in treating schizophrenia.

### 14-3-3-ERK1/2

The M_1_ and M_4_ mAChRs are reported to interact with the adapter 14-3-3 proteins (Borroto-Escuela et al., 2011a). These proteins are most abundantly expressed in the brain and are reported to modulate GPCR trafficking and assemble signaling complexes, such as ERK1/2 signaling complexes (Li et al., 2016; Yuan et al., 2019). The M_1_ mAChR recruits 14-3-3ε-pLuc following stimulation with carbachol (Yuan et al., 2019). For the M_4_ mAChR, validation and characterisation of the potential interaction with 14-3-3 requires further investigation. Characterising mAChR ligands toward 14-3-3 recruitment may be of physiological relevance because 14-3-3 proteins are dysregulated in neurological diseases such as Alzheimer’s disease and schizophrenia (Foote and Zhou, 2012; Graham et al., 2019). This largely unexplored scaffold protein may therefore represent an important novel signaling pathway to target for the development of biased allosteric modulators.

### Phospholipase A_2_

The M_1_ and M_4_ mAChRs are reported to modulate arachidonic acid (AA) levels via activation of PLA_2_. Activation of PLA_2_ by the M_1_ mAChR increases AA release via a PKC-dependent pathway in mouse striatal neurons (Tence et al., 1994), suggesting that this may lead to physiologically relevant signaling. Activation of CHO cells overexpressing the M_1_ mAChR with carbachol increases release of the APP (Emmerling et al., 1993). This secretion of APP is inhibited with the PLA_2_ inhibitors, quinacrine, manoalide and scalaradial, suggesting that M_1_ mAChR-stimulated APP secretion occurs via a mechanism involving PLA_2_ (Emmerling et al., 1993). Inhibition of PLA_2_ signaling may represent an important signaling pathway to target for the treatment of Alzheimer's disease, to reduce the amount of APP that is released from neurons and decrease the burden of accumulation of toxic amyloid plaques. This is supported by evidence from a mouse model of Alzheimer's disease (Transgenic human APP mouse), where increased PLA_2_ activity and AA levels were observed (Sanchez-Mejia et al., 2008). In this mouse model, PLA_2_ was activated by amyloid-β (Sanchez-Mejia et al., 2008). Knockdown of PLA_2_ Group IVA reduced hippocampal AA levels and improved cognition in the Morris water maze test (Sanchez-Mejia et al., 2008). Thus, ligands that can inhibit PLA_2_ signaling via the M_1_ mAChR could have disease modifying utility that could be harnessed for the treatment of Alzheimer's disease.

The M_4_ mAChR can weakly increase AA release when activated by carbachol in CHO cells over-expressing the M_4_ mAChR (Felder et al., 1991). Carbachol stimulation of the M_4_ mAChR also potentiated the adenosine triphosphate (ATP)-mediated release of AA (Felder et al., 1991). In cells pre-treated with pertussis toxin or staurosporine, the AA release was blocked, suggesting that it occurs downstream of PKC and Gα_i/o_ proteins (Felder et al., 1991). Interestingly, increased PLA_2_ activity and AA levels are observed by magnetic resonance imaging of schizophrenic patient brains (Smesny et al., 2010). Thus, compounds that reduce PLA_2_ activity via the M_4_ mAChR may be beneficial in the treatment of schizophrenia.

## Conclusion

The M_1_ and M_4_ mAChRs have the potential to be targeted by new medications to treat the symptoms of neurological disorders, such as Alzheimer's disease and schizophrenia. The challenge in successfully targeting these receptors lays in the selectivity of the ligands and their associated side effect profiles. With the development and characterisation of many new selective compounds for the mAChRs has come the realisation that not all side effects are due to activation of other receptor subtypes, either centrally or peripherally. Instead, some of the side effects are also driven through on-target overstimulation. Biased allosteric modulators offer the potential to overcome these hurdles, through selectively targeting a given mAChR subtype and subsets of its signal transduction pathways within the cell. However, a greater understanding of the pathways and their involvement in the diseases themselves and in the symptoms of the diseases are needed to determine the path forward for developing novel biased allosteric modulators.

In Alzheimer's disease, the M_1_ mAChR is the primary drug target. Studies in genetically modified mice suggest that biasing the M_1_ mAChR to pathways occurring after GRK2 phosphorylation of the receptor would provide relief from adverse effects such as salivation and hyperactivity. Since the M_1_ mAChR is uncoupled from the Gα_q_ proteins in Alzheimer's disease, agonists biased toward β-arrestin pathways may be the most beneficial pathways to target. M_1_ mAChR-β-arrestin-biased modulators may link the receptor to ERK1/2 signaling pathways and improve cognition. Given that overexpression of β-arrestin 2 is linked to increases in amyloid-β secretion, it will be important to avoid overstimulating this pathway. Activation of PLD signaling pathways may also be beneficial, since PLD activation is linked to improved cognition. This could be achieved with ARF-biased or Gα_12/13_-biased ligands, relative to the native repertoire of signaling. However, considering that the Gα_12/13_ pathway is linked to neurite retraction, ligands with bias away from Gα_12/13_ recruitment may be preferred. Compounds that improve M_1_ mAChR coupling to Gα_q_ and away from PLA_2_ may yield Alzheimer's disease modifying benefits, by decreasing APP secretion, amyloid-β production and Tau hyperphosphorylation. Additional benefits may also be seen through targeting the M_1_ mAChR-AKAP79 interaction, by increasing potassium channel activity and cognition.

In treating schizophrenia, the M_4_ mAChR is an attractive drug target, as it is potentially linked to improvements in the positive, negative and cognitive symptoms of the disease. However, the pathways that would be beneficial to target for the M_4_ mAChR are less clear. Based on the currently available evidence, M_4_ mAChR biased allosteric modulators directed toward Gβγ-PI3K-ERK1/2 or β-arrestin-ERK1/2 pathways may improve cognition. Further work is required to identify and characterise the signaling pathways that are activated downstream of the M_4_ mAChR to identify additional pathways that may be beneficial to activate. Treatment of schizophrenic symptoms via the M_1_ mAChR, may require the development of ligands with a more balanced profile of potentiation. This is because the M_1_ mAChR-Gα_q_ pathways are linked with anti-psychotic and anti-anxiety potential. Furthermore, activation of ERK1/2 and PLD signaling pathways via the M_1_ mAChR may improve memory and increase neuronal connectivity, thus directing signaling to specific pathways may lead to the successful development of a new generation of anti-schizophrenic treatments with reduced adverse effects.

## Author Contributions

EW wrote the manuscript and prepared the figures. KC, SM-N, and SB contributed sections regarding mouse studies. CV contributed information for the allostery sections. AT, PS, and AC contributed to the writing of the manuscript. All authors contributed to manuscript revision, read and approved the submitted version.

## Funding

This work was supported by a Wellcome Trust Collaborative Research Award (201529/Z/16/Z), a National and Medical Research Council of Australia program grant (APP1150083) and an Australia Research Council of Australia discovery project (DP190102950). PS is a senior principal research fellow of the National health and medical research council of Australia. SB is a senior lecturer at the University of Glasgow, and supported by a Medical Research Council industrial collaboration agreement (MR/P019366/1).

## Conflict of Interest

The authors declare that the research was conducted in the absence of any commercial or financial relationships that could be construed as a potential conflict of interest.
